# An Historical Essay on the State of the Medical Sciences during the Last Six Months

**Published:** 1823-07

**Authors:** 


THE LONDON
### Medical and Physical Journal.

1 OF VOL. L.] JULY, 1823. [N^O. 293.

For many fortunate discoveries in medicine, and for the detection of numerous errors, the world is indebted to the rapid circulation of Monthly Journals; and there never existed any work, to which the Faculty, in Europe and America, were under deeper obligations, than to the Medical and Physical Journal of London, now forming a long, but an invaluable, series.—RUSH.

### AN HISTORICAL ESSAY
### ON
### THE STATE OF THE MEDICAL SCIENCES
### DURING THE LAST SIX MONTHS.

IN our last Essay, we mentioned that it was not our intention to attempt to give an account, however abbreviated, of all the novelties connected with medical science, which may occur within the periods embraced in our Retrospective Sketches. Further experience has satisfied us of the propriety of limiting our observations to some of the more important subjects; hoping thereby to relieve, in some measure, the dryness which must unavoidably attach to an accumulation of facts, which, from their nature, must for the most part be unconnected. Our desire is, as much as possible, to keep pace with the improvements in medicine and the collateral sciences: with this view we have adopted an arrangement for our monthly INTELLIGENCE, similar to that of our PRÖEMIUM; by which means, many facts, which would otherwise have been noticed here, have already been laid before our readers.

### ANATOMY (NATURAL) AND PHYSIOLOGY.

*Human Anatomy*, for obvious reasons, very rarely presents any new discovery.—Some well-marked instances of the multiplication of parts, as the pectoralis, glutei, and other muscles, have been recorded by Dr. TIEDMANN;* and a more uncommon and important phenomenon, described by the same physiologist, is the occasional existence of a double sexual system in the female, consisting of two vaginæ, each connected with a separate uterus, capable of impregnation independent of the other. These cases, to which we have already referred, (Number for June,) are rather to be regarded as monstrosities, than as coming under the department either of Natural or Morbid Anatomy: they are of importance, however, as affording a new and apparently satisfactory

* *Med.-Chirurg. Zeitung.*

explanation of some of the cases of superfœtation, which rest on authority too unequivocal to admit of doubt.

*Anatomy (comparative).*—A peculiar arrangement of the venous system has been described by Dr. JACOBSON, in the "Isis of Oken," a German periodical publication of some note. In man, and other mammiferous animals, all the veins, with the exception of the vena portarum, are arranged so as to form a single system, which collects the blood returning from the various parts of the body, and conveys it to the heart. The vena cava in this class is formed by the veins from the lower and posterior parts meeting, so as to form one common trunk, which runs directly to the right auricle. Dr. Jacobson has shown that this arrangement does not extend to other vertebral animals; and he describes a new set of veins, which is not directly connected with the general venous system, but which conveys the blood coming from the middle or posterior parts to the kidneys or liver. This arrangement obtains in birds, reptiles, and fishes, being subject to three modifications.

"The first modification, which is to be esteemed the prototype of the rest, exhibits the following form. From the skin and muscles of the middle part of the body branches arise, which form several trunks, passing separately to the kidneys, in the substance of which they again divide into branches, and are there variously distributed.

"In the second modification, the veins which return from the posterior part of the body are received into this separate system, of which we are treating. The caudal vein, which brings back the blood from the skin and muscles of the posterior part of the body, divides into two branches, which, having received some veins returning from the middle part of the body, flow to the kidneys of each side, and distribute their branches in the parenchymatous substance of these glands.

"In the third modification, the veins of this system are formed in the same manner as in the preceding, only that the caudal, or other vein returning from the posterior parts, gives off a branch to the vena portæ. The blood returning from the middle and posterior part of the body in the first and second modification of this system, is conveyed only to the kidneys; but, in the third, to the kidneys and liver. The inferior vena cava of the common venous system, in the second and third modification of this system, is composed of the veins returning from the kidneys and testicles, or ovaries. In the first modification, the caudal vein receives the veins returning from the kidneys, is united with the veins of the testicles or ovaries, and, in this manner, forms the inferior vena cava."*

In fishes, the venous system is found in all its forms,—in many genera presenting the first modification: all the blood from the skin and muscles of the middle part of the body, from the head to the tail, is collected by venous branches, which, uniting into single trunks, run by various routs to the kidneys, and are distributed in their parenchymatous structure. The caudal veins, united to those of the ovaries or testicles, join, or rather form, the inferior cava. The *carp*,

* *Edinburgh Med. and Surg. Journal,* No. lxxiv.

*tench*, *herring*, &c. present examples of this distribution. The second modification, however, is by far the most common; and the third (differing from the second only in this, that the caudal vein, besides the veins going off to the kidneys, also gives a branch to the vena cava, so that the blood goes partly to the kidneys and partly to the liver,) prevails in all amphibious animals. In birds, the veins are arranged according to the last variety; and in these animals the transition to the mammalia may be discovered, since the venous system of Dr. Jacobson unites with the common one. The caudal, ischiatic, and crural veins, run to the kidneys, and give off a large anastomosing branch to the vena portarum: the crural vein, however, likewise sends a branch to the vena cava; so that, in this class of animals, all the blood returning from the posterior part of the body is carried partly to the kidneys, partly to the vena portarum, and partly to the vena cava.

The physiologist above mentioned is convinced, from many experiments upon living animals, that the venous system described by him is intended to regulate the function of secretion. The vena portarum has long been regarded as affording an example of venous secretion; but from this it would appear that, in birds, reptiles, and fishes, the secretion of the kidneys likewise is effected by means of veins and venous blood.

Mr. PATRICK HILL, surgeon in the navy, has described the spur of the *ornithorhynchus paradoxus*.* He observed, near the convex side of the spur, a small spot, like the orifice of a tube; and, on trying to pass a bristle, three drops of a clear limpid fluid issued from it. The other spur was examined, with the same result. On dissecting the foot, he found, at the inner side of the root of the spur, immediately over the articulation, a small cyst, which did not at the time contain any fluid, but from which a horse-hair readily passed through the spur.

*Physiology.*—The researches of M. FLOURENS are, in a physiological point of view, among the most important which have occurred within the period of our Essay; and we beg leave to refer to the Report by CUVIER, which we gave at length in our last Number. At present we content ourselves with a condensed account of the conclusions at which he has arrived.

"According to M. Flourens, there are two properties essentially distinct in the nervous system; the one to excite muscular contraction, the other to perceive impressions. The object was to determine experimentally what parts of this system serve exclusively for sensation; and what, on the contrary, serve exclusively for contraction.

"It is evident that the trial of each part could alone ascertain its property. M. Flourens has therefore subjected to trial, separately and in turn, the nerves, the spinal marrow, the medulla oblongata, the tubercula quadrigemina, the cerebellum, and the cerebral lobes. From these experiments, thus chalked out, it follows—

"1st. That the nerves, the spinal marrow, the medulla oblongata, and

* *Transactions of the Linnean Society*, vol. xii. part ii.

the tubercula quadrigemina, are capable of exciting muscular contractions.

"2d. That the cerebral lobes and the cerebellum are not capable of exciting them. Haller and Zinn had formerly noted the impassibility (insensibility) of the upper layers of the cerebral lobes; Lorry, that of the corpus callosum; M. Flourens has, for the first time, observed this insensibility in the whole of these lobes, in the cerebellum; and has been the first to fix the limit at the tubercula quadrigemina.

"The irritation of a nerve, separated from the nervous centres by section or ligature, is confined to the excitement of abrupt and partial contractions in the muscles to which this nerve is distributed. The nerve, therefore, excites properly only contractions.

"The spinal marrow being cut successively above the posterior enlargement, above the anterior, and near to the occiput: at first the animal lost the use of its hind paws, then of its fore paws, and next of the trunk; but, in all these cases, all these parts, the hind and fore paws, as well as the trunk, preserve their collective movements, *(mouvemens d'ensemble.)*

"We ought to add, that these movements take place only in consequence of external irritations. What has disappeared is, first, the co-ordination (consentaneity) of the movements in leaping, flying, walking, standing, catching, &c.; and, secondly, the volition of these movements.

"What remain are the contractions, and the connexion of these contractions in associated movements. The spinal marrow, then, properly ties the muscular contractions, in associations *(mouvemens d'ensemble)* as to volition and the co-ordination of these movements that resides elsewhere.

"The irritation of the spinal marrow constantly occasions violent convulsions: its destruction speedily brings on death; but this last effect depends on its action on the involuntary movements. Constantly, the abstraction of one of the tubercula quadrigemina causes the sight of the opposite eye to be lost. The irritation of a tuberculum determines contractions in the opposite iris; its complete removal abolishes the contractions completely. In the tubercula, therefore, the primary principle of the action of the iris and of the retina resides.

"In proportion as we cut off the cerebellum by successive layers, the animal loses gradually the faculty of flying or running; then that of walking; and, finally, that of standing upright.

"A single cerebral lobe being removed, the animal loses immediately the sight of the opposite eye; but the contractility of the iris of this eye continues, notwithstanding the animal experiences at first a weakness much more marked on the opposite side of the body. In other respects, it goes on as usual. The two lobes being removed, there is no longer any vestige of volition, or of memory, or of any perception: memory, volition, perception, reside then in the cerebral lobes."

The papers of Mr. Shaw and Mr. Broughton, in our Number for June, will be read with great interest by those who take a part in the investigations so actively carried on, during the last twelve months, with regard to the functions of certain classes of nerves. Mr. Shaw gives a clear and satisfactory description of the nerves which, according to the system of Mr. BELL, are called *superadded;* and one advantage possessed by this new arrangement, which must be acknowledged even by those who deny its accuracy, is that of facilitating, in a remarkable degree, the labour of the student in acquiring a knowledge of this complicated piece of anatomy. Mr. Broughton has detailed various experiments, the chief results of which are, that the portio dura and par vagum are entirely insensible, while the fifth pair is gifted with a high degree of sensibility.—We trust that further reference to papers so lately published is unnecessary.

In a former Number, we alluded to the paper by Mr. C. BELL, read before the Royal Society: through the kindness of Mr. Shaw, we are enabled to give a more particular account of the author's views.*

Mr. Bell has entered into an examination of the external apparatus and muscles of the eye, with the view of explaining the necessity of six nerves being given to the parts contained in the orbit.

In the course of this examination, he shows that the six muscles which are attached to the eye-ball do not, as has been supposed, form one class of voluntary muscles; but that, while the four straight muscles, or recti, are provided for the voluntary motions of the eye when directed to objects, the other two, called *oblique*, perform certain involuntary motions. These involuntary actions are shown to be a provision for the better protection of the eye; for, when the eye-lids wink and close to wash the cornea, the effect would be incomplete, and the object but imperfectly attained, unless the cornea was also at the same time raised by the revolving of the eye-ball.

After having proved that the eye-ball revolves so as to carry the cornea upwards during the motion of the eye-lids, and having also shown that the oblique muscles are the agents in this involuntary and instinctive motion, he proceeds to demonstrate that the same muscles elevate the cornea during sleep.

The author says that, while we are awake, the eye is under the active influence of the four straight muscles; but, when the eye-lids are closed in sleep, these muscles resign their office, and the involuntary oblique muscles prevail so as to draw the cornea under the upper eye-lid. This is also shown to be the condition of the eye in faintness, and on the approach of death, and on all other occasions when languor or debility prevail over the voluntary muscles of the frame.

The author notices incidentally, that the enjoyment of the sense of vision is attended with the excited condition of the recti, or voluntary muscles, and that insensibility to the impression on the eye is followed by relaxation and neglect of the same class of muscles; and, consequently, that a depraved or injured condition of the retina is one cause of squinting; for the oblique prevailing, while the recti, or voluntary muscles, are neglected, draw the eye so affected from the parallel line of vision.

After having shown that the recti, or voluntary muscles, are strictly associated with the activity of the retina, or organ of vision, he proceeds

* MS. notes received from Mr. SHAW.

to express his opinion that the ideas received through the eye are not limited to the office of the retina, but that the sense of vision, properly so called, is aided by the sense of voluntary exertion in the recti muscles; and that these afford us the knowledge of the position and relation of bodies, in addition to the ideas of form, shades, and colours, which are received through the retina.

The paper is illustrated by references to comparative anatomy, and by observations and experiments on man and brutes.

After having described the variety of actions performed by the muscles of the eye, the author proposes, in the second part of the paper, to arrange the nerves which go into the orbit, according to their offices."

Drs. LAWRANCE and COATES continue their experiments upon absorption, with a view of determining the powers of the veins and lymphatics.* They began by further trials with colouring matters, as prussian blue and indigo. These were attended with negative results: none of them entering the vessels, although so much as nine ounces and a half of the former substance was swallowed by a bitch. The greater number of experiments, however, were made with prussiate of potass, as being both easy of absorption and of subsequent detection by tests. A list is given of thirty-four animals, in which this salt was introduced into the alimentary canal; the parts being left untouched, in the first twenty-one, till after the animals' death; in the others, ligatures were applied. The general result is calculated to show that absorption took place principally through the vena portæ, only one example being mentioned in which the colour of the fluid in the thoracic duct was as intense as in the serum of this vein. Our limits prevent us from being able to allude to these experiments more fully, but the following remarks will, we trust, be read with interest:

"The general weight of evidence in these cases is strongly in favour of the principal absorption having taken place through the vena portarum. Only one case is mentioned in which the colour in the fluid from the thoracic duct was not less intense than in the serum of the vena portarum.

"In this instance, the former was not taken until thirty minutes, and the latter in twenty-one; during the interval of which there is every possibility for much of the absorbed substance to reach the points at which both were examined; as much greater diversity than this exists in many of the results. Hence it may be supposed that absorption would have taken place in the porta to a greater extent, had both been examined at the same time. Another circumstance which affects the inference to be drawn in a very material degree, is, that the vena portæ conveys so much larger an amount of fluid than the thoracic duct, that an equal intensity of colour implies the presence of a much larger quantity of the chemical agent. This is also a reply to a suggestion made in the report to the Academy of Medicine, in favour of the thoracic duct as a route. But, as this was based, as far as relates to the

* *Account of some further Experiments to determine the absorbing Power of the Veins and Lymphatics.* By I. O'B. LAWRANCE, M.D. and B. H. COATES, M.D.—(*Philadelphia Journal*, No. 10.)

mucous membranes of which we are treating, upon only seven experiments, and in none had we then proceeded to examine the serum of the vena portarum, it is hardly necessary to array them in opposition.

"Inferences, however, of a more decisive kind may be drawn from some of the experiments which ensue. Five are first enumerated, in which the vena portarum was secured by a ligature. In the two first, the cardia being undisturbed and the fluid introduced down the œsophagus, the œsophageal and pharyngeal veins had access to it, and their radicles or capillaries may have absorbed the salt. A degree of uncertainty also prevails whether the vena portarum was in all these instances properly secured. In the three last, however, this point was carefully ascertained by subsequent dissection, and a ligature was also passed round the cardia to prevent the regurgitation of the fluid into the œsophagus itself. The prussiate was then introduced through a wound in the upper part of the duodenum, and this part also tied. In the first case, the prussiate was detected in the heart in thirty-four minutes; in the second, in thirty-nine; and in the third, in thirty-five minutes. This we consider as proving, directly and decidedly, that there are other means of absorption besides the veins. We now proceeded to tie the thoracic duct, and endeavour to ascertain whether the prussiate could be made to enter the circulation, by passages independent of this. The three first experiments recorded are not quite definite, from the cardia not being secured, as the fluid was liable to regurgitate into the lower part of the œsophagus; a circumstance which we always found to take place when that part was not artificially closed. It may also be remarked, by the way, that the œsophagus was always found, when examined for that purpose after feeding, to contain a portion of the substances swallowed, whenever these retained the fluid or semifluid form.

"As, however, the agent was conveyed into the systems of these animals, it certainly follows, from the two first cases, that another route than the thoracic duct admitted of the passage of the salt. In the last of the three experiments, both this vessel and the trunk of the lymphatics in the right side of the neck were secured; thus stopping every known outlet to the system of lacteals and lymphatics. The blue was, nevertheless, easily produced in the serum of blood taken from the right side of the heart, in twenty minutes.

"In tying the lymphatic outlets, great care and much time were employed. The sufficiency, however, of the ligatures was proved by extreme turgescence of the trunks and all the visible branches, immediately after the application of the ligatures, generally followed by much enlargement, and frequently by their rupture in different places.

"In the next animal, after securing these parts, the cardia was also tied, thus confining the visible means of absorption to branches of the vena portarum alone. Injection of the prussiate was made through an opening below the pylorus, and the wounded part tied. In thirty-two minutes, blood was taken from the right side of the heart, the serum of which gave a strong blue. We regard it, then, as evinced, first, by the two first of these experiments, that other means of absorption than the thoracic duct exist; secondly, by the third, that other routes exist than either that or the lymphatic trunk of the right side,—thus confining them, of all visible vessels, to the sanguineous ones alone; and thirdly, by the last, that absorbed fluids are carried through the trunk of the vena portarum individually, as access was barred to the branches of any other vein.

"In the four next instances, after tying both the two lymphatic trunks and the vena portæ, injections of prussiate of potass were made down the œsophagus, without tying either the cardia or pylorus. In all these cases the prussiate was conveyed into the circulation. In the first, it was discovered to be in the right side of the heart in thirty-six minutes; in the second, in forty-eight minutes; in the third, it was found in the aorta in thirty minutes; and in the last it was exhibited, more faintly, in the shorter period of twenty-five minutes, in the right side of the heart.

"In four cases which follow, all these vessels were first secured, and then the cardia. The upper part of the duodenum was lastly secured, after injecting the agent through it. In none of these was a distinct blue to be found, in either the serum of the right side of the heart or in the urine. In the second instance only a *bluishness* is mentioned as having been visible in the serum of the right side of the heart. As, however, none of the others are so,—as the term is so weak,—and as the urine in the same case, although twenty-five minutes had elapsed, did not indicate it, it affords no very formidable exception. The intervals at which the examinations were made, for the heart, are twenty-five, upwards of twenty-three, twenty-five, and twenty-six minutes; for the urine, thirty-two, upwards of twenty-three, thirty-six and a half, and twenty-eight minutes.

"A comparative experiment was made by tying all the attachments of the stomach whatever, in order to ascertain the effect of simple infiltration. No prussiate was found in any of the fluids, although the stomach, almost immediately after tying, gave an evident blue on applying the test to its outer surface, the animal being alive. The contents of the carotid were removed for examination in thirty-one minutes; of the right side of the heart, in thirty-four; of the bladder, in thirty-seven; and then the pelvis and papilla of the kidney were examined.

"We conceive we have thus established, that articles taken into the stomach may escape by three outlets for absorption,—namely, the vena portæ, the œsophageal veins, and the thoracic duct; and, if all these are closed, the absorbed matters are no longer conveyed to the circulation or to the urine. With regard to the quantity conveyed by each, we have no sufficient means of judging. As the quantity of fluid, however, contained in the vena portarum is so much greater than in the thoracic duct, it follows that, to produce a colour of equal intensity, a much larger amount of the colouring matter is requisite. * * *

"In reasoning upon the subject of absorption, the question has frequently arisen whether the articles found in the living fluids exist there as chemical substances, or have their chemical nature altered and animalized by the action of the vessels through which they have entered the system. In other words, it has been inquired whether the chemical results we obtained were produced without previously causing the death of the fluid, and thus again reducing it to the influence of chemical laws, from which its vitality had previously entirely protected it. The instantaneous changes which take place in the recent chyle on applying the test, seem to forbid the idea of two successive alterations being produced, and one of them commonly so gradual in its progress as the extinction of life. It was, however, deemed a curious subject of inquiry, whether artificial chemical changes can take place in the fluids while they continue to circulate in living vessels, and the ordinary actions of life go on? We can hardly consider fluids as having undergone a change from life to death, while they continue to permeate the living organs, including the brain, and all the functions continue with no greater disturbance than naturally ensues from doing so great violence to the system as is necessary to the experiment. We commenced by throwing prussiate of potass into the abdomen, and green sulphate of iron into the cellular tissue, in order to try whether the well-known result of their admixture, the prussian blue, would be produced in the vessels. This, however, did not take place; and we resolved to repeat it, by throwing the sulphate, as the article of more difficult absorption, into the abdomen, (where this process went on with more facility,) and the prussiate into the cellular substance. On performing this, we were gratified by the striking result of a distinct and beautiful blue in the thoracic trunk and its contents, and in nearly the whole substance and surface of the *lungs*. These viscera were preserved in spirits, and are now in our possession. The blood threw up a coagulum of a strong blue colour, and the lymph and chyle from the thoracic duct threw down a blue deposit. Thus not only a foreign, but a pulverulent substance, could present its unnatural stimulus and circulate through the vessels, and could accumulate in the lungs, without preventing the actions of life from considerable exertion, and without occasioning coagulation of the blood. The animal manifested some difficulty of respiration before she was killed, but walked about without the least difficulty, and uttered no cries, nor other signs of disturbance of its powers. In another case, the urine and lungs are noted in our Journal as exhibiting a blue. The other parts, similar to those above enumerated, are not described as being found coloured. In a third, the fluid in the thoracic duct was blue; but not the other fluids examined, nor the lungs. Two unsuccessful trials were also made. In another case the thoracic duct was tied, and the same process repeated. A decided bluish green was here found in the urine; but neither the serum of the arterial blood nor the lymph of the ductus thoracicus manifested the blue or green. Several inferences may be drawn from this experiment, with which we shall not now trouble the reader.

"We repeated the celebrated experiment of Majendie, in which he separated a limb from the body, except by the double attachment of either an artery and a vein, or of their two columns of blood circulating through quills. We employed nux vomica, and succeeded entirely in one case without the quills, and in two in which they were used. In six other cases, two of which were with prussic acid, we failed. In conducting this distressing operation, we have not escaped the mortification of disappointment: it has, however, been gratifying to us, as far as we went, to verify the results of this enterprising physiologist. They depend, however, on *symptoms* for their evidence; chemical proof of the presence of nux vomica not being capable of exhibition. In one experiment, while waiting the result of an introduction of nux vomica, made ten minutes previously, two drachms of the solution of prussiate of potass, which we employed, were forced into the cellular substance of the separated limb, from a pointed syringe. This salt was afterwards detected in the body, after having passed through one quill with the column of venous blood; thus rendering visible its actual transition, and confirming the results of Majendie."

A paper, recently read to the Institute, contains the result of various interesting experiments on *absorption* and *exhalation*, by M. Fodéra.* The object of this physiologist is to show that exhalation, which he denominates *transudation*, and absorption, which he calls *imbibition*, are in reality the same phenomenon, arising from the imbibition of different vessels, operating in the first case from the interior of the vessel outwards, and in the second from the exterior inwards. Majendie had been led to conclude that venous absorption was effected by imbibition; and one of the experiments leading to this opinion was that of insolating a portion of a venous trunk, and placing its surface in contact with a poison: the presence of this within the vessel was soon manifested. M. Fodéra has reversed this experiment. He injected a poisonous substance, with all necessary precaution, into a portion of artery confined between two ligatures, and insolated, not only from the cellular texture, but likewise, he informs us, from the lymphatics and *vasa vasorum:* the poison took effect. He obtained the same result on filling a portion of an artery, vein, or intestine, with the poison; removing them, and placing them either in a wound made in another animal, or in the abdominal cavity. The rapidity of the poisoning in these cases varied according to the age and species of the animal, the thickness and length of the portion of vessel or intestine used, its more or less complete distention, and the degree of solubility of the poison. The same phenomena presented themselves when sulphuretted hydrogen was employed.

If an artery or vein be laid bare in the living animal, an oozing is observed to take place through the coats of the vessel. This oozing is increased if a ligature be applied, and dropsy may be produced in this manner. From these facts, M. Fodéra concludes that exhalation is only a transudation through the vascular parietes; and many physiologists thought so before the existence of exhalent vessels was suggested.

In a former Number,† we detailed various experiments of M. Fodéra, tending to prove that, in the dead body at least, the transudation of liquids may take place at the same time from the interior to the exterior of the coats of vessels or intestines, and *vice versa.* Without recapitulating what was then laid before our readers, we proceed to notice some further observations by the same author. Phenomena similar to those just detailed are said to present themselves in the living body. M. Fodéra, for example, has found in the bladder, or in the thorax, substances injected into the peritoneum; and, in the abdominal cavity,

---

* *Journal de Physiologie,* Janvier 1823.

† April, 1823.

substances introduced into the bladder or thorax. In these experiments he employed a solution of galls and the sulphate of iron, or else this last and the prussiate of potass. The change of colour into black or blue in these experiments, announcing the occurrence of transudation, was not observed in general under an hour, but it may be rendered almost immediate by employing galvanic influence.*

The conclusions at which M. Fodéra arrives from the result of his experiments, are, "1st, that exhalation and absorption take place by transudation and imbibition, and that they depend upon the capillarity *(capillarité)* of the textures; 2dly, that this double phenomenon may take place in every part, and that the liquids which they have imbibed may be conveyed equally by lymphatic vessels, arteries, or veins." If the effects of absorption have not been manifest in those experiments where a portion of intestine containing poison was insolated so far as to communicate with the rest of the body only by a lymphatic vessel, M. Fodéra attributes it to the extreme slowness of the circulation of the lymph. He placed liquid prussiate of potass in the subcutaneous cellular tissue of the thigh and belly of two young rabbits; and detected this substance, in the former animal at the end of some minutes, and in the second at the end of half an hour, in the lymph of the thoracic duct, in the urine, the mucus of the intestines, the synovia, the serum of the blood, the water of the pericardium, of the pleura, and peritoneum, as well as in all the solid parts, except the crystalline lens, the substance of the brain, the interior of the nerves, and the bones. Now, although these results seem calculated to prove that absorption takes place both by lymphatics and blood-vessels, yet it is necessary to keep in mind that the mere presence of a substance in a lymphatic vessel does not constitute absorption. "That absorption may be effected, (says M. Majendie,†) it is not sufficient that the sides of a vessel imbibe, but the substance must also be carried along towards the heart: imbibition and transportation constitute absorption."

M. Fodéra has likewise devoted considerable ingenuity and research to the explanation of the phenomena which attend the rapid passage of various substances from the stomach to the bladder, by which it appears that the experimentalists of this country were mistaken in supposing that such transition was effected by some other than the ordinary medium of the lymphatics or blood-vessels.—M. Fodéra introduced a catheter, with a cork adapted to it, into the bladder, and then injected a solution of prussiate of potass and iron into the stomach: as soon as the salt was detected in the urine, (an occurrence which in one instance took place in ten, and in another at the end of five minutes,) the animals were instantly opened, and the prussiate was found in the blood of the vena cava inferior, of the heart, and of the aorta, in the thoracic duct and other parts. These experiments, if found by others to yield similar results, must be admitted as proving both the extreme rapidity of absorption, and that the communication between the stomach and bladder must be looked for in the usual course of the circulation.

---

* See the Number for April. † *Journal de Physiologie*, Jan. 1823.

The celerity with which absorption is effected in some organs, is rendered still more striking by experiments upon the lungs. M. Fodéra injected prussiate of potass into the trachea, and cut out the heart of the animal (a rabbit,) as soon after as possible. The operation was performed in twenty seconds; and, notwithstanding the shortness of the time, the interior of the left auricle was stained of a greenish-blue colour, which was deeper in the mitral valve, and less apparent, although still perceptible, in the aorta. It will be observed that the results detailed by M. Fodéra agree, in general, with those obtained by Majendie.

A memoir of considerable interest, on the *exhalation and absorption of azote in the lungs*, has been published by Dr. EDWARDS.* The results of experimentalists differ very widely on this subject: some, as Humboldt, Spallanzani, Davy, &c. believed that a notable quantity of azote was absorbed during respiration; Allen and Pepys, that no sensible change of its quantity occurred; and lastly, Berthollet and Nysten found it to be increased, instead of diminished. It is the opinion of M. Edwards, that in the different experiments, where diminution of the quantity of azote takes place on the one hand, and increase on the other, there are two views which may be taken of these results. In the first, the quantity of azote which disappears would be regarded as arising from absorption alone, and its increase to exhalation: only one of these functions takes place at the same time. In the second, the two functions of absorption and exhalation may be regarded as going on simultaneously; and, consequently, the results would only give the difference in their action. Thus, when an animal breathes in the atmospheric air, these two functions being simultaneous, azote would be absorbed on the one hand, and exhaled on the other; and, from the relation between the absorption and exhalation would necessarily arise three different results, according to circumstances. When the exhalation exceeds the absorption, exhalation alone would be the apparent effect; when the absorption predominates, the converse would hold good; and, lastly, when the two functions are in the same proportion, no obvious effect would result, but the azote expired would just equal that inspired. Upon this principle is attempted the explanation of the jarring opinions upon this subject.

In our last Number will be found the detail of numerous experiments by Dr. WILLIAMS, intended to prove the existence of some sources of inaccuracy in the conclusions formed by Dr. CARSON, with regard to *the effects of wounds made into the chest.* We beg to refer to the interesting paper of Dr. Williams for his reasoning upon this subject, while we direct our attention to another attack upon the accuracy of Dr. Carson, made in a late American publication.†

This gentleman, after the performance of various experiments, came to the following conclusion, " that the difference of the distribution of

* *Annales de Chimie et de Physique*, Janvier.

† *Experiments and Reflections on the Cause of the Vacuity of the Arteries after Death.* By WILLIAM FENNEL, M.D. of Virginia.—(*Philadelphia Journal*, No. 9.)

the blood after death from that in which it must have existed in the living system, arises chiefly from the elastic power of the lungs; and that the emptiness of the arteries, and of the smaller vessels, observed after death, admits of a satisfactory explanation from the supposed operation of this cause, combined with that of the elasticity of the arterial canals." Dr. Fennel informs us that he was led, by various circumstances, to doubt the accuracy both of the experiments of Dr. Carson and his inferences; he proceeds to say:

"Exactly after the manner of Dr. Carson, I took a half-grown rabbit, and made two incisions, one on each side, between the fifth and sixth ribs, an inch in length. In six minutes the animal expired from a collapse of its lungs; and as soon as it ceased to breathe its body was opened, which gave the following appearances:—The heart still continued to act, though very imperfectly. The arteries could be seen to diminish; while the veins, more particularly the large veins, were enlarged. When the left auricle and ventricle were opened, they contained very little blood. I placed two ligatures around the aorta, one at its great bifurcation, the other a few inches above it, and cut out the enclosed part: on examination, it was found to contain a small quantity of blood. The cutaneous arteries and veins could be seen very beautifully anastomosing, when I first separated the integuments from the body, which in these animals is very easily accomplished, even without the aid of a scalpel. In the space of five minutes the arteries were empty; and the veins, though not so much dilated as they were at first, contained blood enough to make them round.

"On cutting into the muscles, no blood exuded, as is asserted by Dr. Carson; nor were the muscles more red than in the animals killed by cutting the spinal marrow. The membranous parts exhibited the blood-vessels very much like the skin, and in a few minutes their arteries contained no blood; their veins were round, but not as large as when I first opened the body of the animal. The intestines gave nearly similar appearances, and, after the cessation of action in the arteries, they showed the same phenomena as did the membranes and cutaneous parts. The liver was of a reddish-brown colour. On opening the right side of the heart and pulmonary artery, with the venæ cavæ, there was an inundation of blood, partly coagulated: the pulmonary veins were quite empty, and the lungs of a light red colour.

"After a repetition of these experiments, sufficiently often to satisfy us that in every instance the same phenomena would take place, I cut the spinal marrow of other animals, near the superior cervical vertebræ, which produced death in seven minutes. I opened the bodies as in my first experiments, and found in every instance the same appearances, except in the pulmonary veins, which contained a small cylinder of partly coagulated blood; and the lungs, which were larger and of a deeper colour than in the former experiments, owing to the greater quantity of blood they contained.

"Having repeated these experiments sufficiently often to convince all present of their accuracy, I killed several animals by collapse of the lungs, also some by cutting the spinal marrow as before, and suffered them to lie several hours before I opened them: on examination, no difference could be found as respects the state of the arteries."

Having thus demonstrated, to his own satisfaction, and that of his coadjutors, that the appearances presented by animals killed by collapse of the lungs agree in all respects with those exhibited by animals put to death by dividing the spinal marrow, Dr. Fennel concludes that there is not any tendency in the lungs to form a vacuum ; or, if such exist, it does not drain the other parts of the body of their blood; but, on the contrary, this physiologist offers the following explanation of these phenomena:

"That respiration ceases previously to the entire cessation of the action of the heart and arteries, in animals which are killed or die a natural death, is a fact that every one must acknowledge. This being the case, when respiration ceases the lungs become flaccid, and thus occasion a partial obstruction in the weakened circulation. But the heart and arteries continuing to act sufficiently to empty themselves of the blood imposed upon them, forcing it into the veins, which tubes being less powerful than the arteries, and having the obstruction in the lungs to oppose the passage of blood from the right to the left side of the heart, they unavoidably become the part of the circulatory system which must contain the great mass of the blood after death.

"That the partial obstruction in the lungs, and the contraction of the heart and arteries, are the cause of the vacuity of the arteries, is proved, as well by my own experiments as by the appearances on dissection of animals killed by lightning. The life of the animal here being destroyed in an instant, the arteries, not having the power to contract, cannot throw the blood from their cavities into the veins; on which account the blood of animals thus destroyed is always found in the arteries and veins in a just proportion, though the elasticity in every part be the same as before death."

Some interesting observations on *the effects produced by bile in the process of digestion*, have been made by Mr. BRODIE.* As we have not before mentioned these to our readers, we shall give them in the author's own words.

"When an animal swallows solid food, the first change which it undergoes is that of solution in the stomach. In this state of solution it is denominated *chyme*. The appearance of the chyme varies according to the nature of the food. For example, in the stomach of a cat, the lean or muscular part of animal food is converted into a brown fluid, of the consistence of thin cream; while milk is first separated into its two constituent parts of coagulum and whey, the former of which is afterwards re-dissolved, and the whole converted into a fluid substance, with very minute portions of coagulum floating in it. Under ordinary circumstances, the chyme, as soon as it has entered the duodenum, assumes the character of *chyle*. The latter is seen mixed with excrementitious matter in the intestine; and, in its pure state, ascending the lacteal vessels. Nothing like chyle is ever found in the stomach ; and

* *Journal of Science,* No. xxviii.

Dr. Prout, whose attention has been much directed to the chemical examination of these fluids, has ascertained that albumen, which is the principal component part of chyle, is never to be discovered higher than the pylorus. Now, in my experiments, which were made chiefly on young cats, where a ligature had been applied so as to obstruct the choledoch duct, the first of these processes, namely, the production of chyme in the stomach, took place as usual; but the second, namely, the conversion of the chyme into chyle, was invariably and completely interrupted. Not the smallest trace of chyle was perceptible, either in the intestines or in the lacteals. The former contained a semi-fluid substance, resembling the chyme found in the stomach; with this difference, however, that it became of a thicker consistence in proportion as it was at a greater distance from the stomach; and that, as it approached the termination of the ileum in the cæcum, the fluid part of it had altogether disappeared, and there remained only a solid substance, differing in appearance from ordinary fæces. The lacteals contained a transparent fluid, which I suppose to have consisted partly of lymph, partly of the more fluid part of the chyme, which had become absorbed.

"I conceive that these experiments are sufficient to prove that the office of the bile is to change the nutritious part of the chyme into chyle, and to separate from it the excrementitious matter. An observation will here occur to the physiologist: If the bile be of so much importance in the animal economy, how is it that persons occasionally live for a considerable time, in whom the flow of bile into the duodenum is interrupted? On this point it may be remarked, 1st. That it seldom happens that the obstruction of the choledoch duct from disease is so complete as to prevent the passage of the bile altogether; and the circumstance of the evacuations being of a white colour may prove the deficiency, but does not prove the total absence of bile. 2dly. That in the very few authenticated cases which have occurred of total obliteration of the choledoch duct in the human subject, there has been, I believe, always extreme emaciation, showing that the function of nutrition was not properly performed. 3dly. That the fact of individuals having occasionally lived for a few weeks or months under these circumstances only proves that nutrition may take place to some extent without chyle being formed. In my experiments I found that the more fluid parts of the chyme had been absorbed, and probably this would have been sufficient to maintain life during a limited period of time."

### MORBID ANATOMY AND PATHOLOGY.

*Morbid Anatomy.*—Many individual cases of great interest are to be found in the various periodical works of the last few months: these it is impossible for us to notice individually, and we are therefore constrained to limit our remarks to more general questions.

Much attention has been devoted by Dr. Baron, of Gloucester, to the examination of *tuberculous diseases*, and in a late publication* he has advanced some new opinions with respect to the progress of tubercles

* *Illustrations of the Enquiry respecting Tuberculous Diseases.* By J. Baron, M.D. &c. &c.

of the lungs. In the descriptions of these by the most distinguished pathologists of modern times, they have been represented, when first perceptible, as little bodies of various size and consistence. Dr. Baillie compares them to the heads of " very small pins; and M. Laennec informs us that they vary " from the size of a millet-seed to that of a hemp-seed :" both these eminent men agreeing that tubercles, when they can be first perceived to exist in the form of little granular bodies, possessing such a degree of consistence as to render them capable of being distinguished from the surrounding textures by the sense of touch. This, if not expressed, is distinctly implied in the descriptions of all morbid anatomists with which we are acquainted. Here, then, is the first feature of novelty in the work before us; Dr. Baron maintaining that tubercles, when first formed in the lungs, are not to be detected by the touch, on account of their extreme delicacy and the elastic nature of their structure. " They are (says he,) very small vesicular, transparent bodies, and shine amid the unchanged texture of the surrounding lung." He subsequently compares them, when arising on the surface of the membranes, to the globular incrustations to be seen on the leaves and stalk of the ice-plant. There is nothing inconsistent, we would observe, between this description and that which precedes,—except that the former begins at a later period of the disease. Our author acknowledges that, in the human subject, these tubercles are very rarely seen in this early stage of their progress; that the size is speedily increased, the transparency diminished, and the general character of extreme delicacy lost; in short, they now assume that granular form which others have described as their first perceptible stage. That such a phenomenon as that described by Dr. Baron may occasionally exist, and yet have escaped the scrutiny of preceding anatomists, we can conceive quite possible: first, because it is very rare,—that is, persons very rarely die in so early a period of phthisis; and, secondly, from the difficulty that there must necessarily be in determining their existence. They are represented as extremely minute, perfectly transparent, and vesicular: reflecting upon these characters and the peculiar texture of the lungs, we cease to wonder that bodies of such delicacy should have escaped notice.

In a dissection lately performed at La Charité, in Paris, by MM. Andral and Breschet, the lungs were found filled with hydatids, some of which also were situated in the pulmonary veins. " Many of these hydatids were lodged in pouches having a smooth surface, which appeared to us at first so many cysts; others, empty and rolled up many times upon themselves, were contained in narrow canals, of which they had taken the elongated form. The internal surface of these canals was smooth, like that of the large pouches: they ramified like vessels. Finally, we soon discovered that there terminated at each pouch a vessel of small calibre, which, in order to form it, became more or less considerably dilated. We then dissected the pulmonary veins to their origin, and, when we had arrived almost at their capillary division, we began to see many of them present a great number of dilatations, which were filled with hydatids. After having undergone this enlargement, the vein resumed its former dimensions, and then became dilated again a little further on. The larger pouches would have contained a large nut, while the smaller would scarcely have admitted a pea. They existed equally in both lungs. The hydatids which they contained had all the characters of *acephalocysts.* Many presented in their substance small points, of a white appearance, and others showed a number of miliary granulations on the internal surface. The greater number were broken. The pulmonary tissue round them was partly sound and crepitating; in others deeply gorged, and even hepatized."*

One of the most important contributions to pathological anatomy which has for some time appeared, is the Essay on the *digestive canal,* by M. ANDRAL.*—M. Andral commences his interesting observations by remarking that the alimentary canal, when inflamed, is for the most part contracted, and appears injected if viewed externally: such contraction, however, is by no means a certain indication of inflammation, as it is not unfrequent about the pyloric extremity of the stomach and in the great intestine, without evidence of any increased vascular action. At the same time he warns us, that the state of the mucous membrane can never be judged of from the external examination, as he has frequently found it inflamed, and even ulcerated, where the peritoneal covering has been free from disease. The commencement of inflammatory action in the mucous coat is attended by an increased flow of blood to the part; and M. Andral has been in the habit of detaching the membrane in this and other stages of disease, to examine its separate state. Separated, then, from the rest of the intestine, and held between the eye and the light, the mucous membrane, under these circumstances, appears either covered with a fine net-work of vessels, with interstices between them, or else these are so close as to give the appearance of a general stain or dye pervading the whole. The colour, however, is by no means invariably red, but, on the contrary, frequently presents a deeper or lighter shade of brown. This does not depend upon the duration of the diseased state, so much as upon its violence, and may be produced within an hour by the introduction of certain corrosive poisons into the stomach of animals. This brown colour is, to a certain extent, indicative of incipient disorganization, and is generally attended with softening. The injection of the part with blood must, of necessity, be productive of thickening; and in chronic diarrhœa it has been known, by M. Andral, to acquire four times its natural dimensions. This thickening may be either general or confined to a small portion: when circumscribed, it forms round or longish patches raised above the surrounding level, and varying in size from a five-franc piece to that of a franc. These are sometimes found in the stomach, but are more common in the colon, and most common of all in the inferior part of the small intestine: sometimes they are solitary, and in other

* *Journal de Physiologie,* Janvier 1823.

† *Sur l'Anatomie Pathologique du Canal Digestif, consideré dans sa Portion sous Diaphragmatique.* Par M. ANDRAL, fils.—(Nouveau Journal de Medecine, Novembre 1822.)

cases occur in great numbers. They likewise vary in colour; some being red, and others white: the former are conjectured to have been of recent formation, and the latter to have resulted from inflammation, the other consequences of which have passed away.

Besides the thickening and soft or pulpy state of the mucous membrane, M. Andral likewise describes what are called *vegetations*, consisting of elevations of a red or brown colour, rising four or five lines above the surrounding level, extremely soft, and bearing some resemblance to the papillæ of the tongue. These he has only found in the great intestine; but a case is mentioned by Orfila, where they occurred in the stomach of a man who had taken powdered cantharides two months previously. Pustules are likewise described: these are conical, generally depressed in the centre, and white, so that they bear a great affinity to those of small-pox; which they also resemble in being grouped. They are found most frequently in the lower portion of the small intestines. In the colon, pustules of a different kind from those described are frequently present: they are described as resembling boils, having a bare and pointed head. M. Lerminier proposes calling them *exantheme interne*. This is one of the morbid alterations frequently found in fatal cases of fever.

The inflamed state of the membrane lining the alimentary canal gives rise to a corresponding change in the secretions: the mucus, instead of being tenacious and consistent, becomes liquid like serum, and in other instances is inspissated so much as to form a false membrane. This is generally found in the stomach or great intestine. In a girl, twelve years of age, M. Andral found a number of inflamed patches on the inner membrane of the stomach, and each of these covered by a layer of grey tenacious matter, resembling, or rather constituting, a false membrane; which, however, did not extend to the other parts of the stomach, which were not inflamed. Sometimes inflamed portions of bowel contain a reddish liquid effusion, which appears to consist of blood and mucus. Sometimes the quantity of fluid poured out is immense; Morgagni mentions an instance of a woman voiding by stool so much as forty pounds in a day. It is also asserted by M. Andral, that, when a portion of mucous membrane is inflamed, it excites an increased flow of bile towards it.

In such cases as we have been describing, the cellular substance immediately beneath the mucous membrane generally remains healthy, even when the inflammation runs high; but in chronic cases it frequently assumes a dense structure, and sometimes is very much thickened.

The muscular coat does not seem subject to many alterations of structure: it is sometimes so soft as to be easily torn, and sometimes assumes a schirrous degeneration; but the most frequent occurrence in inflammation of the neighbouring parts, is to find the muscular coat contracted. This is easily shown by injecting any corrosive poison into the intestines. M. Tartra mentions the case of a person who died three months after swallowing nitric acid: the intestinal canal was so much contracted that the calibre, throughout the whole extent, was no larger than that of a quill. These violent and irregular contractions are regarded as one of the causes producing into-susception; a state which, though frequently combined with inflammation, more commonly exists without it.

Proceeding from within outwards, according to the order of M. Andral, we arrive at the sub-peritoneal cellular substance, which is but very seldom affected: when, however, it does participate in the diseased action, the change of texture chiefly consists in fragility and increased thickness. The peritoneum very seldom participates in inflammation originating in the mucous membrane: when, however, this is the case, it becomes easily torn; and occasionally the whole parietes of the intestine becomes so soft as to be lacerated by the slightest force, or even reduced to pulp by rubbing in the hand. This change is more frequent in the stomach than elsewhere.

Ulcerations are next mentioned, and are supposed to arise from the previous disorganization: they may occur in any part of the tube, but are most frequent in the ileum, and least so in the duodenum and rectum. When they exist in the stomach, they are generally limited to one or two, but in other parts of the canal they are numerous. In the upper part of the small intestine they are always distinct from each other, but in the lower portion they are often blended together, forming a large ulcerated surface. Papulæ and pustules have been mentioned as occurring in the mucous membrane of the intestine; and, as on the tops of these a small eroded point may be perceived, there is little difficulty in supposing this to extend gradually towards the base, and so give rise to ulcers, in the same manner as papulæ and aphthæ of the mouth. The size of these ulcerations varies very much; some are scarcely so large as the head of a pin, and others extend several inches in every direction. Some are of an oblong shape, some exactly circular, and others linear. In the cœcum, M. Andral has found the mucous membrane entirely removed by ulceration, for more than six fingers' breadth. The edges (which are always formed by the mucous membrane) are sometimes elevated, and sometimes they are on a level with the base of the ulcer; sometimes they are red and thick, sometimes white and thin; and, lastly, they are occasionally irregular, giving the appearance of fringes. When the ulcers are numerous, the membrane between them is sometimes quite detached from the subjacent cellular texture. If the ulceration be of recent formation, the laminated tissue at the bottom is of a natural appearance; but, if it be of some standing, it acquires such a degree of thickening that it may be felt by the finger externally; it becomes uneven and granulated; it is of a red, brown, or greyish colour, and secretes a fluid which sometimes passes into the form of a false membrane; lastly, it may be black, resembling an eschar. The most general process, however, by which these ulcerations are formed is that of insensible absorption, laying bare the muscular tunic: this sometimes retains its natural state; in others, it is destroyed in its turn, the ulcer extending to the peritoneal coat. This last membrane next becomes implicated, and perforation of the intestine results.

Such is the progress of ulceration when it extends in depth; but, in the great majority of cases, it merely spreads along the inner membrane. Perforations, however, may take place without ulceration, where all the coats of the intestine have become softened at the same time: the force exerted by its contents may, under such circumstances, rupture the bowel.

When perforation has supervened, it may produce a communication between the alimentary canal and the external surface, constituting artificial anus; or between the canal and some other organ, as the liver, kidney, or bladder; or, lastly, the opening may communicate with the peritoneal sac, and the contents of the intestines be effused into it. Patients are sometimes made aware of this having happened, by some peculiar sensation at the moment of its occurrence. This accident proves rapidly fatal, frequently within a few hours. The general consequence of effusion into the peritoneum, is the speedy occurrence of acute inflammation: occasionally, however, the inflammation assumes the chronic form; and a curious instance is mentioned by M. Andral, in which a preternatural opening was formed, by which the contents of the intestines were evacuated. A young man, labouring under pulmonary consumption, was affected with diarrhœa, unaccompanied with pain: at length he complained of great uneasiness about the umbilicus, which was increased by pressure, and continued for eight or ten days. At the end of this period the patient felt the abdomen wet with a yellow liquid, and perceived a fissure or rent at the umbilicus: during the same day, an intestinal worm (*ascaris lumbricoides*) was voided by this new anus. Another circumstance is mentioned, as sometimes preventing the bad effects which must otherwise arise from perforation of the intestinal canal,—viz. the application of some part so accurately to the ulcerated opening as to prevent it from being pervious. Thus, M. Andral has seen one of these apertures covered by the folds of the mesentery, and also the spleen accurately adjusted, although without any adhesion, to an opening in the great curvature of the stomach. It has been asserted that effusions from the intestines into the peritoneum have been found to exist, without having excited inflammation; but M. Andral conjectures, apparently with justice, that these supposed perforations must have been lacerations, produced in the course of the dissection: an opinion which derives still further probability, when we remember the degree of softening which occasionally results from inflammation.

Suppuration is another result of inflammation, and generally takes place on the free surface of the membrane; sometimes, however, it occurs in the cellular texture beneath, resembling the abscess of cynanche tonsillaris. Such submucous collections of matter are extremely rare; the best example mentioned by M. Andral occurred in the duodenum. He has likewise several times seen small white spots formed by a pearly fluid, which changes its situation on running the finger over the membrane covering it.

Gangrene of the intestines is regarded by M. Andral as of much less frequent occurrence than has been heretofore supposed, although in some diseases, as typhus, it is not unusual to find true eschars on the mucous membrane, which, by dropping off, give rise to ulceration: sometimes these ulcers are found with the eschar nearly detached, and only adherent by a thin shred. These appearances are compared to what is seen on the surface of blisters when they run into mortification; and it is further asserted that the formation of these eschars on the surface of the skin and mucous membrane of the intestines seem to coincide,—an opinion which, if correct, might lead to important practical results.

It appears, then, that there are three stages of this inflammation: first, there is merely a greater or less degree of injection of the mucous membrane; secondly, an alteration of its structure, consisting in thickening, softening, or an exanthematous condition; and, thirdly, disorganization and ulceration. The two latter are so well marked as scarcely to admit of doubt, but not so the former; for, as the simple engorgement of blood which occurs in the lungs immediately before death may be confounded with pneumonia, so the mere existence of an unusual quantity of blood in the mucous membrane of the bowels may be mistaken for inflammation.

In order to avoid this error, M. Andral enters into the following considerations.—Some hours before death, when the action of the venous blood towards the heart has experienced some considerable interruption, the intestines are found to become more or less injected. The obstruction, whether existing in the heart or lungs, causes the blood to fall back upon the liver, which likewise becomes loaded, and refuses to admit the blood coming from the vena portæ: and hence the injection of the intestinal canal. This congestion, while it still remains purely mechanical, may exist in various degrees. In the first, or slightest degree, the vessels of the submucous cellular tissue become gorged with dark blood, giving a marbled appearance to the stomach, and forming dendritic delineations on the intestines. When the accumulation goes a little further, minute branches are observed, highly injected, and passing into the mucous membrane, which sometimes presents brownish-red patches; and occasionally true ecchymosis of the cellular texture occurs. Lastly, the mucous membrane, when the engorgement is at its height, suffers the blood to ooze from its surface: this sanguineous exhalation is at the same time observable in other parts, as the lungs, bronchiæ, and brain. This appearance may likewise be artificially produced, by placing a ligature on the vena portæ, or by putting animals to death slowly.

*Tubercles* are very frequently met with in the cellular texture beneath the mucous membrane, particularly in the lower end of the jejunum and in the ileum. They are of a white colour, and vary in size from the head of a pin to a pea; they are for the most part insolated, and seldom become united, as in the lungs. Sometimes they are completely covered by the mucous membrane, but more frequently ulcers are found at the same time, the bottom of which consists of the remains of the tubercles. M. Andral is of opinion that these formations are independent of inflammatory action, although marks of inflammation are frequently to be seen in the part where they exist. A great analogy between these and pulmonary tubercles is pointed out by the author, who says that they are present in the majority of patients labouring under consumption of the lungs. Another form of tubercle is the millet-seed or miliary granulation: these may be detected, from their hardness, by running the finger on the surface. They are likewise transparent, and are developed in the texture of the membrane with which they may be separated from the subjacent tissues.

Although the stomach and rectum are the portions of the alimentary canal in which tubercles are least frequent, yet they are the parts where the cancerous and medullary degenerations are most common. When these become considerable, no trace of the muscular coat remains, and the mucous membrane, having become disorganized, forms the ragged edges of an ulcer, the base of which is formed by the softened cancerous tissue. In a few cases, the cancer commences in the mucous coat itself, which becomes soft, thick, and ulcerated; sometimes it forms a fungous excrescence, united by a stalk to the mucous membrane.

Another diseased structure of these parts is described under the appellation of *erectile tissue*. These are small roundish bodies, of a brown colour, connected to the internal surface of the bowels by a pedicle, and which exude fluid blood of a dark colour. On a more minute examination, they are found to consist of a set of filaments intersecting each other, so as to leave interstices of various form and size. These tumors are rare, and, when they occur, only one or two are found in the whole canal.

M. Andral next describes what he calls *melanosis of the intestines:* this consists of small black tumors under the mucous membrane, about the size of a nut. In the small intestines an immense number of black spots is sometimes found, covering the mucous coat to the extent of several feet: these exist in all conditions of this membrane, and M. Andral thinks that they are not accompanied by any morbid symptom.

M. Andral maintains the existence of œdema of the intestines, which was denied by Bichat.

*Pathology.*—Many seem to have some difficulty in perceiving the difference between Morbid Anatomy and Pathology, using these terms indiscriminately. To such we recommend the perusal of Dr. PRING's "Exposition,"* lately published. This work is the only one expressly on Pathology which has appeared within the last six months, and consists of an able and learned, but abstract, and perhaps too elaborate, examination of the leading doctrines which have at different times constituted the systems of the schools, together with original views and illustrations of various pathological principles. The nature of this essay prevents us from being able to enter upon so wide a field, but we shall take a future opportunity of recurring to the subject.

Dr. CRAIGIE, of Edinburgh, has devoted considerable attention to the pathology of the *brain*, and the principal facts which he has recorded are to be found in our last volume. In a continuation of his paper,† he insists at some length upon the connexion subsisting between morbid affections of the brain and diseased conditions of the heart and great vessels. We believe it will not be new to our readers in general to learn that organic mischief at the centre of circulation proves a

* *An Exposition of the Principles of Pathology and the Treatment of Diseases.* By DANIEL PRING, M.D. &c.

† *Edinburgh Med. and Surg. Journal*, January 1823.

frequent cause of apoplectic seizures; but, at the same time, this connexion has not been pointed out so distinctly by preceding writers as to render the observations of Dr. Craigie superfluous. The conclusions at which he arrives are as follows:

"1st. It is quite obvious that several maladies of the heart, such as ossification of the left side, or of the artery connected with it; ossification of the mitral valve; of the semilunar valves; arctation of the apertures, either auriculo-ventricular or aortic, have a tendency to terminate in extravasation within the cranium, producing apoplexy, paralysis, or a comatose state terminating in death.

"2d. It is equally obvious that, although examples of disease of the head, affection of the brain and its membranes, occur spontaneously, yet not a few are the result of a disturbed or irregular state of the circulation, induced by disease of the heart or its appendages. This connexion has not been examined so often as it merited; and there is strong reason to believe that, if made the subject of particular investigation, it would be proved to be much more constant and uniform than has been anticipated.

"3d. It is by no means difficult to see how these effects in the cerebral organ result from an irregular and disordered action of the heart. The difficulty which the blood experiences in passing either, 1st, through the auriculo-ventricular opening, 2d, the aortic orifice, 3d, along the aorta, necessarily produces a stagnation and congestion,—1st, in the pulmonary veins; 2d, in the pulmonary artery; 3d, in the right side of the heart. The effect of this is to retard or impede very remarkably the return of the blood from the cerebral veins, and consecutively either to distend them, or, unless they are remarkably strong and resisting, to rupture them, or to occasion an effusion of the serous part of the blood, as we find in other examples of obstructed venous circulation."

That *tubercles*, if they be neither numerous nor large, may lie dormant in the lungs for a longer or shorter period, is an opinion advanced by most writers; but Dr. Baron carries this idea further,* and supposes that these bodies, when once they are fully consolidated, do not subsequently pass into a state of suppuration, and, of course, that such consolidation is to be desired as a favourable termination of the disease.

"The consolidation, therefore, just referred to may, in some measure, be considered as a favourable termination to tubercle, as life has been found to be compatible with their existence, except in cases where they occupied a large proportion of the lung, or produced accretion of the membranes. It is in that period of their progress which is intermediate between the state last mentioned and their first development, that all the symptoms characteristic of tuberculous phthisis occur. This will be apparent by attending briefly to the ordinary progress of the disease.

"In a person who has tubercles, a frequent cough without any expectoration, but with occasional oppression about the chest, and hurried respiration on slight exertion, may exist at intervals for many months,

* *Illustrations, &c.* quoted above.

or even a longer period, without any other sign of disease. What is commonly called a fresh cold may increase these symptoms and render them more permanent, and then the patient, who never expectorated before, may perhaps be surprised by spitting up a yellowish or whitish globular shaped mass, tinged with blood, or a gush of blood may precede an occurrence of this kind. I have known the last-mentioned symptom repeatedly happen, to a most alarming extent, in a case where there was great destruction of the pulmonary tissue by the consolidation of tubercles, but where, though the case proued fatal, there was never any expectoration of the matter from tubercles. It is from this and other kindred cases that I infer that tubercles, once consolidated, do not subsequently suppurate or ulcerate."

This doctrine, our readers will at once perceive, is diametrically opposite to that advanced by M. LAENNEC, who, while he admits the incurability of phthisis in its early stages, is convinced "that, in some rare cases, the disease is curable in the latter stages; that is, after the *softening* of the tubercles, and the formation of an ulcerous excavation:" and again, "The tubercles of the lungs differ in no respect from those situated in the glands, and which, under the name of scrofula, after being softened and evacuated, are often followed by a perfect cure."*

Some cases have been related in which the *thoracic duct* has been more or less obstructed, but the only case in which the obliteration was complete, with which we are acquainted, is to be found in a recent German publication;† and, as such cases must be regarded as extremely rare, even should we be mistaken in regarding the present one as unique, we shall detail the particulars.—A workman, fifty-eight years of age, became a patient at the hospital at Bonn, in March 1821, under the care of Dr. NASSE. He laboured under dropsy: his countenance was expressive of great anxiety; his respiration oppressed, and accompanied with cough; the pulsation of the heart was soft and feeble; the appetite was impaired, and the thirst increased. The belly was tense, and fluctuation was perceptible; the urine scanty and high-coloured, and the hypogastric region painful; the stools scanty; and the lower extremities much swelled. Two months before his admission, this patient had experienced chills, with fullness about the hypochondria, and tendency to vomit. The swelling began at the pubis, extended over the rest of the abdomen, and was soon followed by œdema of the legs. The water, amounting to twelve pints, was evacuated by puncture: tonics and sudorifics were administered, with but little benefit. The belly began to swell again, with pain, increased by pressure, stretching from the pubis to the umbilicus; the skin was loaded with a dry, itching, papular eruption. The appetite increased, while the emaciation and debility continued to advance. Eight days before death, no pulse could be felt in the left arm, which was paralyzed, and this side was more swelled than the other; lastly, diarrhœa supervened, and he died on the 25th of April. On opening the body, the pyloris was found to

* FORBES's Translation of LAENNEC, pages 17, 43, and 44.

† *Leichenoeffnungen; zur Diagnostik und Pathologischen Anatomie.* FREDERICK NASSE, M.D.

be scirrhous, the liver much enlarged, and the viscera of the thorax and abdomen in general loaded with white tubercles. After removing the contents of the chest and abdomen, a cord, of the size of a goose-quill, was observed situated on the bodies of the vertebræ, and ascending from the lumbar region into the thorax, between two rows of tumors: this cord terminated at the junction of the left subclavian and jugular veins, from which it was inferred to be the thoracic duct. There was no trace of its canal left, the whole interior being filled with a white mass; the coats were thickened, but the valves could be distinguished, still retaining much of their natural appearance. As there are none of the symptoms which may not be accounted for by the other morbid appearances, without reference to the state of the thoracic duct, we fear no practical inference can well be deduced from this case. Dr. Nasse seems rather disposed to attribute the disease to venereal origin, an idea altogether improbable.

Dr. Scoutetten, of Metz, has published some Anatomico-Pathological Researches,* the object of which is to show that an intimate connexion exists between irritation of the mucous membrane of the alimentary canal and the pia mater, which he denominates *meningine*, after the nomenclature of M. Chaussier. He endeavours to account for the fact of this mutual connexion not having been more insisted upon by practical writers, upon the supposition of their overlooking the diseased states of this delicate membrane, from the changes not being very conspicuous to a superficial observer. In its natural state, he informs us that the pia mater is perfectly transparent, thin, colourless, and without adhesion; and, consequently, whenever this is not the case, that it must be diseased. Here, at least, the remarks of Dr. Scoutetten deserve attention, as there are doubtless minute deviations from healthy structure which require an experienced eye to detect them; and his recommendation that we should bring into immediate comparison the parts supposed to be diseased with similar parts known to be healthy, cannot but be regarded as desirable wherever it is possible: this was the method adopted by M. Wenzel in examining the brain of epileptic subjects.—According to Dr. Scoutetten, all parts of the intestinal canal do not sympathise to the same extent with the meninges: thus the stomach and small intestines possess a closer affinity with them than the great intestines. And, further, he supposes that the various coats of the intestines possess relations to the head entirely different: thus, while inflammation of the mucous membrane gives rise to corresponding action in the pia mater, this last remains free from all sympathy in peritonitis, although so severe as to prove fatal. When the mucous membrane of the stomach or small intestines is inflamed, the pia mater has its vessels injected with blood, forming red patches, and sometimes giving rise to extravasation. These appearances, according to Dr. Scoutetten, are chiefly found in the anterior and lateral parts of the brain, unless the abdominal inflammation be very violent, in which case nearly the whole of the *meningine* corresponds in its action,—its minute vessels carrying

* *Journal Universel des Sciences Medicales*, Decembre 1822.

red blood, giving rise to effusion and adhesions. The author likewise thinks that his views are calculated to explain many of the phenomena of apoplexy, on the principle of every inordinate excitement of the stomach being communicated to, and as it were repeated in, the brain; thus occasioning determination of blood to the head, and rendering the vessels gradually weaker, and more ready to be ruptured by increased action.

The state of the *digestive canal* in diarrhœa, dysentery, and lientery, has received important illustration from the researches of M. ANDRAL, fils.

"These affections (says he,*) have been for a long time looked upon as diseases entirely independent of inflammation of the intestines. Many ancient authors have, in truth, spoke of the ulcerations seated at the internal surface of the digestive tube in chronic diarrhœas, but they consider them as an effect of the diarrhœa. Such was the opinion of Boerhaave, and of Van Swieten, his commentator. We have already seen that such also was pretty nearly the idea of Stoll; and it may be likewise found in the writings of Hippocrates. He was not ignorant that in dysentery the intestines are the seat of more or less deep ulcerations, but he regarded them as being occasioned by degenerated bile and phlegm.

"Is relaxation of the bowels constantly connected with an inflammatory condition of the mucous coat of the intestines? This is a very important question in a therapeutical point of view. We shall endeavour to answer it by presenting a summary of the numerous cases which we have collected on the subject.

"We have several times found, in individuals who had laboured under recent or chronic diarrhœa, the internal surface of the intestinal canal very pale in its whole extent, the mucous coat having preserved its thickness and ordinary consistence. Patients weakened by long organic diseases, hydropics, old people under that state of debility denominated by the ancients cachexia, and who succumbed after having laboured, for a longer or shorter time, under considerable looseness, frequently present that state of the intestinal canal. Their stools are copious, very liquid, purely watery; surpassing by much the quantity of liquids taken in. We have sometimes found, in cases of this kind, a well-marked serous infiltration of the sub-mucous cellular tissue.

"Morgagni has transmitted to us the history of several cases of diarrhœa, without inflammation of the mucous coat of the intestine. He has seen several individuals labouring under that disease die in a short space of time, exhausted by the excessive abundance of their alvine evacuations. In these atonic diarrhœas, the intestinal parietes frequently become much extenuated; the fleshy coat, especially, experiences a true atrophy, and occasionally seems to be only composed of some pale and small fibres, widely separated from each other. Bonet had already remarked this fact. 'In chronic diarrhœa, (says he,) we

---

* *Nouveau Journal de Medicine.*—We avail ourselves of the translation in our respected cotemporary, the Medical Repository, Number for June.

find the intestines as thin as a cobweb.' The intestine, in this state, seems to be unable to fulfil its functions: chylification is but imperfectly performed, absorption becomes much less active, and the food is frequently voided as when taken. This is what the ancients designated under the name of *lientery*.

"The mucous coat of the intestines may then, like many other textures, become the seat of a much more abundant secretion than usual, although it presents no trace of inflammation. It is thus that, during convalescences from chronic maladies, the exhalation of serosity into the subcutaneous cellular tissue is frequently augmented. It was not, therefore, without reason that Sauvages designated under the name of *flux* a particular class of diseases.

"Since truly atonic fluxes may exist, it follows that a strengthening and astringent treatment is, in these circumstances, the only proper plan to be pursued. Thus the œdemata, of which we shall presently speak, may be dispersed, either by the employment of topical stimulants, or by the internal administration of tonic medicines.

"In other individuals, we find the mucous coat of the intestines equally white in its whole extent; but beneath it numerous tubercles, or other adventitious tissues, exist. They provoke diarrhœa, either by the sympathetic irritation which they determine to the mucous membrane covering them, or by stimulating by their presence the muscular coat, the contractions of which consequently become more rapid and more intense. It is in this manner that the different adventitious tissues, developed in the parenchyma of the lungs, provoke an habitual irritation of the mucous membrane of the bronchiæ; but, most commonly, the diarrhœa in this case does not appear to become permanent and considerable, until the period when the tubercles, having become softened, inflame and ulcerate the mucous coat.

"It is moreover indubitable, that, in a very large majority of cases, the intestines of individuals labouring under diarrhœa, whether complicated or not with dysenteric symptoms, present evident marks of phlegmasia.

"This phlegmasia may be seated in the small or in the large intestine.

"In the small intestine, it frequently only exists to the extent of some fingers' breadth above the ileo-cæcal valve; at other times a larger portion of the small intestine has laboured under it, where it either appears under the form of a simple injection of the mucous coat, alteration of its texture, red or white softening of its tissue, or ulceration.

"Numerous cases have apprised us that acute or chronic diarrhœa is the frequent result of isolated inflammation of the small intestine, without the large participating in it in any manner. We insist on this fact, because M. Broussais has laid down, as a general principle, that enteritis is accompanied with constipation, and that diarrhœa only supervenes when enteritis is complicated with inflammation of the colon *(colite)*.

"Of the three portions of the great intestine, the cæcum is that which, in diarrhœa, most frequently presents one of the three degrees of inflammation; after that the colon; and, lastly, the rectum.

"The symptoms, the *ensemble* of which constitutes dysentery, are not connected with any particular state of the intestines. The tenesmus alone announces that inflammation exists in the rectum. As for the bloody and glairy stools, they appear in individuals whose intestines present lesions analogous to those which are observed in other patients whose stools had always been purely watery.

"We once found a somewhat considerable number of ulcerations in the ascending colon, in a phthisical patient, who, after having previously laboured under diarrhœa, had not felt it for a long period, and had even become constantly constipated. It may be conceived that this may be the case when the ulcerations are small, by no means numerous, and when neither the edge nor the base is inflamed. In fact, at such times they can only, like tubercles, produce a flux by the sympathetic irritation of the mucous coat surrounding them, or of the muscular coat.

"The different states which the digestive tube may present in diarrhœa being well known, can they be distinguished during life from the symptoms which manifest themselves? This, in many cases, is possible. Thus, if pains of the abdomen are observed,—if the skin is burning hot, the pulse frequent,—if the dejections are slimy, membraniform, or bloody, we may be satisfied that the intestine is the seat of more or less intense inflammation.

"We may add, however, that nothing is more common than the absence of every species of pain in cases where numerous ulcerations cover the internal surface of the intestines, whether of the ileum, cæcum, or colon. How frequent also is it, on the other hand, to see patients complain of violent pain in the abdomen, although the digestive mucous coat is not the least inflamed. Is not this the case, as the success of the treatment confirms, in lead-colic, which is cured by the employment of the most active drastics,—in the colics termed nervous, which frequently yield to eminently stimulant medicines,—and in those which are owing to accumulation of flatus and of fecal matters, and which are treated with so much advantage by repeated purgatives? Stoll has cited a remarkable case of syphilitic intermittent colic, which yielded to the use of corrosive sublimate.

"We have already seen that intestinal tubercles may arise, become developed and softened, without any pain indicating their presence.

"The character of the stools is not always itself a certain sign by which we can recognise inflammation. Sanguineous evacuations have been observed to take place *per anum*, in individuals whose intestinal mucous coat was found sound after death. These passive hæmorrhages are analogous to those which take place in many dropsical individuals, at the internal surface of the serous membranes of the chest and abdomen; they are similar to the hæmorrhages of which the skin, the cellular tissue, and the synovial membranes, become the seat in scorbutics.

"The serous dejections, resembling water, coloured yellow or green, manifest themselves equally in all possible conditions of the digestive tube,—in the cases where it is ulcerated, and in those in which its parietes are pale, thin, and œdematous *(infiltrees)*.

"When even ulcerations exist in the intestines, ought they to be regarded as a constant obstacle to the employment of tonic and astringent substances? They present such a great variety in their nature, that it seems the same method of treatment cannot agree with all.

"The white, grey, or brown colour of the base,—the nature of the secretion which takes place there,—the want of, or the considerable thickening of, the laminated tissue which forms it,—the appearance and disposition of their edges,—the different degrees of consistence, of thickness, and of colour, of the mucous coat which constitutes them,—the separation of this membrane to a greater or less extent,—its state in the spaces between the ulcerations,—are they not so many circumstances which seem to demand a multiplicity of modification in the treatment? We may thus easily explain how any curative method succeeds very well in one case, and completely fails in another. We have seen, for example, several diarrhœas yield to the decoction of catechu; we have seen others increase, and become aggravated, during the administration of this medicine, although in both cases the symptoms were nearly the same, and the patients placed in nearly similar general circumstances: the major part were consumptive individuals. It would frequently be of great importance, could we, in the same portion of intestine, apply an astringent or tonic substance upon the ulcerations, and cover with emollient applications the spaces which separate them, and reciprocally. In this manner the surgeon acts in the treatment of several ulcers situated on the surface of the body. He heals them by endeavouring to keep up inflammation to a certain degree, above and below which the disease cannot proceed towards resolution. Is it not, again, by the employment of topical astringents that many chronic ophthalmiæ are cured? Is it not, also, by the employment of resinous substances that chronic phlegmasiæ of the mucous membrane of the lungs and urethra may be very successfully treated? We have very frequently seen M. Lerminier have recourse, with marked advantage, to a slightly stimulant treatment towards the end of acute pneumonia, having a tendency to pass into the chronic state.

The account given by Mr. TRAVERS* of the symptoms which occurred in the late Dr. Pett, from a wound received in dissection, will be read with much interest. This gentleman had assisted in examining the body of a lady who died of peritoneal inflammation; the wound (situated on the middle finger of the right hand,) was so slight as to escape observation, till the occurrence of pain during the same evening led to a minute examination. It is remarkable that the application of nitrate of silver, and subsequently of strong sulphuric acid, produced no sensation in the first instance, although a second application of the lunar caustic was soon followed by intense pain. Next morning, "the finger was white and without sensation." The accident took place on Saturday, and by Tuesday the arm had recovered its natural state, being neither swollen nor painful; but a considerable effusion had taken place into the axilla and over the pectoral muscle; the swollen part was covered with an erithematous blush, painful, and "crepitated on pressure, as in emphysema." Dr. Pett died 105 hours after the injury

* *London Med. and Phys. Journal,* February.

had been inflicted; great nervous irritability and anxiety having accompanied the progress of the malady. No light was thrown on the phenomena by the post-mortem examination.

This case bears a considerable analogy to those related by Dr. COLLES, in the third volume of the Dublin Hospital Reports; an account of which is to be found in the 48th volume of this Journal.*

The *effects of putrid matters*, when applied to the living body under different modifications, formed the subject of an interesting essay by Dr. GASPARD, in the second volume of the Journal of Physiology.—Similar experiments, and with similar results, have more lately been performed by M. MAJENDIE,† who succeeded in producing, within a few hours, various maladies similar to those resulting in man from exposure to putrid exhalations. Vomiting, for example, and black stools, were produced by the introduction of putrid injections into the sanguiferous system. In pursuing these inquiries, Majendie found that different kinds of flesh acted with different degrees of power during their putrescent state: thus, the muscular fibre of herbiferous appeared much less active than that of carnivorous animals; but, of all substances, the most deleterious was water containing putrid fish,—some drops of this producing, within an hour, symptoms having a great analogy to those of typhus or yellow fever. Death generally took place in twenty-four hours; and, on opening the body, all the traces were found of the blood having undergone chemical change, as it remained in great part fluid, and had transuded, in an unwonted degree, through the different textures, particularly the mucous membrane of the intestinal canal. It is remarkable, although not at variance with the analogy of other poisons, that the same putrid water received into the stomach was entirely harmless; a result which Majendie attributes to the mucus of the parts acting as a filter, preventing the passage of the more solid part of the putrid matter: by filtering the water through paper, much of its virulence was lost. Another form of the experiment consisted in exposing animals to the action of putrid effluvia: some suffered no apparent injury, while others, as dogs, became rapidly emaciated, and died at different periods within twenty days. The prosecution of these experiments may possibly throw some light on points of pathology at present involved in utter darkness.

Various pathological facts seem to show the existence of similar diseases in man and the lower animals, and a new example of this kind has been recorded within the period allotted to our present observations.‡ A person lately presented himself at the Royal Infirmary of Edinburgh, having a sore on his arm, produced, according to his own account, from touching the leg of a horse affected with *farcy*. An ass was inoculated in the leg with the matter from this man's arm, and a

* While this sheet was passing the press, another instance of death from this cause occurred in London: if possible, we shall lay the particulars before our readers in a future Number.

† *Journal de Physiologie*, Janvier 1823.

‡ *Edinburgh Med. and Surg. Journal*, January 1823.

disease was brought on supposed to be farcy, as symptoms of the glanders appeared some days after, and proved fatal. On dissection, the usual morbid changes were found; among others, the ulceration of the septum of the nostrils, regarded as characteristic of this disease.—Many of our readers are probably aware that a veterinary surgeon in this metropolis had the misfortune, a few years ago, to become inoculated by the matter of farcy from a horse, and fell a victim to the disease under circumstances particularly distressing.

### SEMIOLOGY.

No work expressly on this subject has lately appeared, and our remarks must therefore be very limited: indeed, the only disease the symptomatology of which has been the subject of particular investigation, is *puerperal fever*. This has been discussed with much zeal by our northern brethren since the period of our former Essay, although, we regret to say, without such satisfactory advancement of our knowledge as might have been expected. This does not proceed either from want of talent or want of opportunity among them, but because they have not agreed about what is to be called puerperal fever, and what peritonitis, in lying-in women. Until this is done, it is obvious that satisfactory results cannot be attained; for at present it is notorious that what one man calls puerperal fever another denominates peritonitis, and *vice versa*. Our present purpose, however, is not to review opinions, but to state them; and, accordingly, we proceed to give the symptoms which indicate the puerperal fever of Drs. CAMPBELL* and MACKINTOSH.†

The disease, in the great majority of cases, is said to come on within the first three days, and to be ushered in by rigors and other febrile symptoms. This is quickly followed by pain in the abdomen, the exact seat of which has been differently described by various authors. Dr. Campbell says, "In all my cases, there was pain in the hypogastrium at the commencement of the disease, darting into one or both iliac regions. In a few examples of this affection, patients described the pain as having commenced in one or other of the iliac regions, and extended towards the uterus, which organ felt enlarged, and was exceedingly sensible upon pressure. In my practice, therefore, I can with confidence assert that the pain, in the beginning of this affection, was chiefly confined to the hypogastric and iliac regions. Patients never complained of it in the umbilicus or epigastrium, except in one case, until the disorder had continued for some time; and I am firmly of opinion, that those writers who describe the pain as having been chiefly seated in the epigastric region, in some instances, at the commencement of the disease, must have deceived themselves by confounding its stages."

Such is the seat of pain at the commencement; but, if the disease be not speedily arrested, it gradually extends to the umbilicus, and thence

---

* *A Treatise on the Epidemic Puerperal Fever, as it prevailed in Edinburgh, in* 1821-2. *To which is added, an Appendix, containing the Essay of the late* Dr. JORDAN *on the Puerperal Fever of Aberdeen, in* 1789-90-1-2. By WM. CAMPBELL, M.D. &c. &c. &c. 1822.

† *A Treatise on the Disease termed Puerperal Fever, illustrated by numerous Cases and Dissections.* By JOHN MACKINTOSH, M.D. 1822.

into the epigastric region; having intervals of remission, followed by exacerbations of great violence. Even from the beginning, some degree of tumefaction is perceptible about the abdomen, and this increases with the progress of the disease, sometimes until it becomes as prominent as before delivery. The uterus can generally be felt above the pubis, is extremely sensible to the touch, and, according to the opinion of Dr. Campbell, undergoes actual enlargement. The pulse becoming quick is regarded by this author as the first symptom which denotes the threatened evil; and, when the disease is fairly established, it rises to 110, 120, or 130; is sometimes full, but more frequently hard, becoming contracted, and sometimes intermitting, in the progress of the disease. The tongue is generally white and moist, the edges and raphe being sometimes of a fiery red. Nausea prevails in the commencement, and subsequently vomiting; the matter ejected consisting first of phlegm and ultimately resembling coffee-grounds. The evacuations by the bowels vary in appearance, being dark brown, grey, or ash-coloured, generally frothy, and extremely fœtid; the purging is accompanied with severe griping pains. One of the symptoms regarded by Professor Hamilton as pathognomonic, is the presence of the lochial discharge, which he asserts is *never* suppressed in genuine puerperal fever. Dr. Campbell's opinion is thus expressed:

"Some say that there is more or less of a suppression of the lochia in every example of the disease; others, again, that it is altogether suppressed in some cases; while the distinguished Professor Hamilton declares that it does not suffer any change, and that the disease cannot be puerperal fever where the uterine discharge is suppressed. My experience corresponds in some measure with this eminent individual; for in all my cases, except one, the uterine discharge was always present to some extent; and I have since been of opinion, that I had in this instance suffered myself to be deceived by the attendants, who often say that the lochia are suppressed when they really are not. Although this discharge continued to flow, to use the words of a celebrated author, there was always 'more or less of a suppression of it;' and this was particularly conspicuous immediately after the accession of rigors,—a change naturally to be expected, as all the secretions are diminished during febrile excitement, not only in the puerperal state, but on every other occasion. I would not, however, wish it to be understood, that I should be so illiberal towards my brethren as to insinuate that they have allowed themselves to be deceived in all their cases; for I should suppose that there are deviations to be remarked in the lochia, as well as in every other symptom of the disease."

So entirely satisfied is Dr. Campbell of the accuracy of his diagnostic characters in this disease, that he thinks a practitioner ought to be brought under the cognizance of the law for ignorance, should he treat for any other disease than puerperal fever, a lying-in woman who had "acute fixed pain in the lower part of the abdomen, aggravated on pressure, or a general soreness of the abdomen, rendered more acute by pressure; accompanied with frequent pulse, hurried inspiration, and much uneasiness on turning to either side in bed."

### STATISTICAL MEDICINE.

For various reports relative to the diseases prevalent during the last six months, we refer to our preceding Numbers. Probably the most interesting question which occurs in this department of our Essay relates to the state of *small-pox* and *vaccination;* but on this subject we are unwilling to enter. We have already laid before our readers Reports from the National Vaccine Board, and from the Physician to the Small-Pox Hospital.

The sickness which has lately prevailed to such an alarming extent in the Penitentiary at Millbank will probably occupy some of our attention, as soon as the Minutes of the Evidence before the House of Commons have been printed.

Since our review on the subject of the *Barcelona fever* a work has appeared,* which should certainly have obtained a place in our Analytic Department, had we not so recently discussed the bearings of this question. As it is, we embrace the opportunity of laying before our readers the opinions of Dr. O'Halloran on the origin of the yellow fever in the south of Spain; opinions derived from zealous investigation on the spot.

"The occurrences which preceded the appearance of the epidemic of Barcelona in 1821, correspond with the old and recent observations on a similar subject in other countries; it almost invariably happening that the yellow fever of Spain is preceded by unusual diseases of various form and force,—more particularly by bilious remittents, which are not unfrequently so aggravated and malignant that physicians themselves do not venture to define the line of demarcation between them and the avowed epidemic.

"This want of decision, or confession of inability to judge conclusively of the true character of the disease, occasioned considerable controversy in Barcelona. The medical faculty could not correctly ascertain the precise period at which the endemic bilious remittent ceased, and the real epidemic fever began. The difficulty was embarrassing; for, although the question has been the subject of much unpleasant controversy, it is not yet decided to the satisfaction of the public where the difference lies. This uncertainty among physicians, as to the nature of the disease at its first appearance, is no inconsiderable proof of the analogy which subsists between the endemic bilious remittent and the epidemic yellow fever. A fever, bearing some of the diagnostic marks of this last disease occurs annually in this city. The first cases which appeared in 1821 passed unnoticed, or were considered only as the ordinary diseases of the season, and treated as such. A few weeks subsequent to the appearance of these aggravated cases, and when it was discovered that a formidable form of malady prevailed in the place, some of the physicians, and the majority of the people, unwilling to acknowledge that a malady of such malignity had its origin among themselves, conjured up a tale of importation, ascribing to in-

* *Remarks on the Yellow Fever of the South and East Coasts of Spain, &c. &c.* By Thomas O'Halloran, M.D. 1823.

tercourse with the infected subjects of shipping, &c. the sickness and deaths which took place in the month of July.

"The first deaths, according to report, occurred on-board a Neapolitan sloop of war, which anchored in the harbour of Barcelona, on the 23d of April, (a cruiser in the Mediterranean,) and on-board the frigate Liberty, lately from America; at least these were the first noticed by the government: and, according to the prevailing notions of contagion and importation, suspicion attached to the vessels which had lately arrived from the Havannah. Much has been said on the subject of importation in this instance; but no one has attempted to prove the fact by distinct evidence. The probability only is admitted, and the importation has been fixed, by supposition only, on the following vessels:

"1. The brig Talla Piedra, Captain Narcisco Paris, sailed from the Havannah on the 28th of April, 1821, with a cargo of sugar, coffee, logwood, and tobacco; touched at Carthagena on the 12th of June, landed two passengers, and took another on-board. She finally arrived at Barcelona on the 19th of June, where she got pratique, after having performed eight days' quarantine.

"2. Brig Nuestra Senora del Carmen, Captain Don Pablo Soler, sailed from the Havannah on the 28th of April, 1821, with a cargo of sugar, wax, coffee, logwood, and tobacco. This vessel arrived at Carthagena on the 16th of June, obtained pratique; disembarked her second pilot. She arrived at Alicant on the 29th, where she unloaded part of her cargo, embarked a passenger, and, finally, cast anchor in Barcelona harbour on the 11th of July.

"3. Brig Grand Turk, Captain John Segreras, sailed from the Havannah on the 28th of April, 1821, with a cargo of sugar, pepper, coffee, cotton, and bullion. She arrived at Cadiz on the 5th of June, where she obtained pratique; disembarked twenty-four passengers, embarked four others and three sailors; and, finally, arrived at Barcelona on the 29th of June.

"4. The Spanish frigate Liberty, Captain James Sinderas, sailed from the Havannah on the 28th of April, 1821, with a cargo of coffee, cocoa, tallow, rum, and bullion. She arrived at Malaga on the 8th of June, obtained pratique, and disembarked part of her cargo; touched at Carthagena, where she also disembarked a part of her cargo, a passenger, and a sailor; and, finally, arrived at Barcelona on the 28th.

"The above-named vessels are those to which suspicion has been attached, as being the vessels in which the yellow-fever was imported. They sailed from the Havannah in a fleet consisting of fifty-two sail, for the following ports: viz.—thirteen for Cadiz, twenty for Barcelona, six for Corunna, three for Santander, four for Malaga, one for Vigo, one for Ferrol, one for Bilboa, one for Palma, one for Lisbon, one for Bahia.

"According to the creditable testimony of Mr. Bruno Vidal, an officer of the Spanish army, who returned to his native country in a vessel belonging to the fleet in question, the inhabitants of the Havannah enjoyed good health prior to his departure; there being only a few slight cases of the usual fever of the season in the town, and those confined to newly-arrived Europeans, particularly sailors. It appears also, from an official document which I have now before me, that only three deaths occurred in that part of the fleet destined for Barcelona, during the voyage; and that no suspicion has been attached to the vessels in which the deaths took place.

"The history of the disease among the shipping in Barcelona harbour is involved in obscurity, or rather total darkness. There is no clear evidence, nor authenticated fact, to prove the reports in favour of importation; but, notwithstanding this deficiency, the whole of the authorities, a few of the physicians, and the mass of the people, maintain with obstinacy that the disease originated on-board the ships from America, and diffused itself in the town, in consequence of newly-imported contagion. It is stated, as I before remarked, that the disease, according to report, made its first appearance on-board a filthy Neapolitan vessel, lately arrived from some port in the Mediterranean. This is report, but it is not of reliance; for the fact is well authenticated by the British vice-consul, (who resided in the Plaza de la Constitucion, which commands a view of Barceloneta,) and supported by the most eminent physicians of the city, that several deaths took place in Barceloneta and Barcelona, at points distant from the supposed source, as soon, if not prior, to the deaths on-board ship. The disease was not at that time regarded as yellow fever; but the attending physicians now declare that it was analogous to what was afterwards known under that name. The physicians of Barcelona, it may be observed, had not the opportunity of treating the yellow fever before this season; and on that account much controversy arose respecting its character, and the name by which it ought to be designated. It was only on the 14th of August that it was decided to be the yellow fever of America, but not contagious. In consequence of this decision of the Board of Health, the port was lined with troops, all communication with the shipping was cut off, the sick were removed to lazarettos, and the usual precautions against presumed contagion were regularly adopted.

"The authorities, perceiving the slow and gradual progress of the malady, and willing to make the public believe that it was wholly confined to the port, declared in the public papers that they had it within their control, and consequently that it should be brought to obedience. The promise was bold, but not wise. Force of arms was of no avail; the disease started up in different parts of both towns, without a possibility of ascertaining from whence it came. The futility of guards became obvious; the troops were removed from the port, and the gates of the city were closed against the inhabitants of Barceloneta. After that was done, the malady spread with extraordinary rapidity in Barceloneta, and in that part of Barcelona which skirts the port: scarcely an individual escaped who was long exposed to the atmosphere of these localities; for there the morbific cause would seem to have been concentrated in a singular degree. From Barceloneta the disease marched in a westerly course through the centre of Barcelona, diminishing in activity as it proceeded outwards. It was finally lost before it reached the north-west extremity of the city; and, beyond a given limit, the epidemic character ceased, or only appeared by accident.

"The circumstances which lead to the rejection of the doctrine of importation are strong and numerous, in so far as respects the epidemic of Barcelona, as well as in every other. I shall state them in as concise a manner as possible.

"1st. Before the importation of yellow fever from Havannah to Spain can be admitted as probable, it must be proved that there existed at that place a contagious fever, of similar character to the disease in Spain, at the time of the departure of the fleet. Without this, the supposition of importation is a mere gratuitous assumption, contrary to reason, and repugnant to common sense. Yellow, or concentrated endemic fever, I believe, never prevailed at the Havannah in the month of April; the disease commonly taken for it being the bilious remittent, which attacks Europeans, newly arrived, at all seasons of the year, but which rarely, if ever, attacks persons at that season of the year whose constitutions are assimilated to the climate. The form under which this disease appears on such occasions, is generally remittent; sometimes so obscure, indeed, in its periodical movements, as to resemble the concentrated endemic of the autumnal months; but, however dangerous and complicated in its mode of proceeding, it never yet, I believe, has been considered to be contagious,—at least it has not been thought so by experienced men. If this be the case, and the fact is founded upon the authenticated evidence of learned physicians of all nations, it is clear that the material which produced yellow fever at Barcelona could not be imported. It is generally acknowledged that the yellow fever of South America or the West Indies is not contagious at any season of the year, or under any circumstances of management; in confirmation of which opinion it may be necessary to adduce a fact, which proves to a demonstration that the disease is not exportable. There never has been an instance of its propagating in well-ventilated hilly situations in the interior, even at the small distance of three miles from Vera Cruz. The noxious cause is circumscribed to a certain locality. Thousands of instances are on record of persons who contracted the disease in town dying in the country, without a solitary example of its affecting the most assiduous of the attendants. If the disease, then, be not contagious at its origin, how can it be imported, and so changed in its nature as to assume a different character in a foreign country?

"2d. It, moreover, appears extraordinary how it could have happened that the crews of vessels carrying this noisome pestilence survived, and even preserved good health during a long voyage; or how they could, after having touched at different ports, discharge part of their cargoes in places that had formerly suffered from the yellow fever, without a single instance of injury being produced by the intercourse. If the cause of disease were actually on-board, this is inexplicable. Cadiz, Malaga, Almeria, Alicant, and Carthagena, &c. &c. &c. were more exposed to injury from the intercourse which took place between the crews of the vessels alluded to and the inhabitants of the above-mentioned places, than Barcelona, a port situated at the eastern extremity of Spain; yet they escaped.

"3d. Had the disease been an imported contagious disease, it would have propagated in any atmosphere, in any town, and under any condition of climate. Its ravages would not be confined to one spot, as was the case in the present epidemic; for even parts of the infected city were exempt from sickness, where no precaution was taken to guard against its inroad; and numerous villages, towns, and country houses, where individuals, who exposed themselves to the epidemic cause at its source, sickened and died, without detriment to the mass of their neighbours, or to their own immediate attendants. This could not have been the case had the disease been imported as a contagion. It would have retained its original character: the power of propagation, though modified by locality, season, or temperature, would not have been annulled.

"4th. Had it even been proved that a disease prevailed at the Havannah of a contagious nature, at the time these vessels sailed for Europe, and that a portion of the contagion had been embarked on board, it is still evident, even according to the doctrine of the advocates of contagion, that the period between the departure from the Havannah and the arrival in old Spain is tantamount to a quarantine of the most extended duration, with the additional advantage of constant strong ventilation from the high winds which usually occur at sea.

"5th. As it is an undeniable fact, not disputed by the most prejudiced contagionist in Spain, that cold invariably destroys the epidemic yellow fever of that country; and as ships, in their return from the Havannah to the continent of Europe, are exposed to more intense cold than is experienced at the sea-coasts of Spain at any season of the year, it appears unaccountable why a similar effect is not produced by a similar cause; or why excessive cold does not act on the germ of a disease on ship board, which it absolutely extinguishes on shore even in a few days.

"Lastly. The departure from the Havannah of the ships in question with clean bills of health,—the trifling sickness and mortality which took place on the voyage,—the touching at other ports before they arrived at Barcelona, without any suspicion of communicating contagion to those ports,—and, moreover, their speedy admission to pratique at Barcelona itself, are circumstances which still further divest the Havannah ships of every reasonable suspicion of having been the importers of this dreaded malady."

### THERAPEUTICS AND MATERIA MEDICA.

This branch of medical science has been indebted to no writer of the present day more than to Dr. PARIS; and we cannot begin what we have to say on the subject more appropriately than by laying before our readers some of the general views respecting the operations of medicinal substances, which are contained in the last edition of his "Pharmacologia." These, indeed, were published anterior to the period embraced in our present Essay, but unfortunately not early enough for us to introduce them in our preceding "Historical Sketch." Dr. Paris is of opinion that the immediate effect of a remedy may depend upon mechanical, chemical, or vital agency; and, further, that such curative impressions may be either *absolute* or *relative*, *primary* or *secondary*, *local* or *general*, *direct* or *sympathetic*, *permanent* or

*transient.* His opinions are illustrated in the following manner:—Certain medicines, called purgatives, excite alvine evacuations under every condition of the body, and may therefore be called *absolute* agents: diuretics, on the other hand, generally require for their operation a certain favourable state of the system, and are denominated *relative.* The difference between the *primary* and *secondary* operation of medicines is well explained by referring to the class of diuretics: of these, some act primarily, stimulating the secreting vessels of the kidneys by actual contact, such as potass, nitre, turpentine, &c.; while the direct agency of others, as digitalis, is upon the absorbents, the diuresis being a secondary effect. This class of medicines illustrates well the circumstances attending the *local* and *general* action of remedies,—how they may excite distant organs by entering into the circulation, and being thus brought into contact with them; or they may excite a local effect upon the stomach and bowels, "thus arousing their energies through the mysterious medium of sympathetic communication." While many medicinal substances undergo digestion, yet, according to the experiments of Dr. Paris, there are others which pass through the stomach unchanged, and, entering the general system either by the vena portarum or thoracic duct, are subsequently brought into direct communication with particular organs. This is said to hold good more especially with regard to many alkaline salts, which, having passed through the kidneys, exciting them to increased action, may afterwards be detected in the urine by chemical tests. Various essential oils, bitter principles, and colouring matters, are capable of resisting digestion, and circulating unchanged to remote parts. In other instances it would appear that compound bodies are sometimes decomposed, one ingredient being digested, and the other passing unchanged: such, for example, is the potassæ acetas, the vinegar of which is acted upon by the stomach, while the alkali enters the circulation. There is still another change which the digestive organs are capable of exerting on medicinal substances,—viz. their entire decomposition, by which they may be rendered wholly inert. Dr. Paris imagines that this may be the reason why substances exciting great energy upon some animals, are comparatively inert when exhibited to others. Those habituated to vegetable food are less affected by vegetable poisons than carnivorous animals, so that a dog is destroyed by half the quantity of opium which a rabbit might take with impunity.

The action of remedies by sympathy, through the medium of the stomach, is too obvious to require illustration: that the stomach, however, is not the only organ capable of propagating sympathetic impressions, is demonstrated by the dilatation of the pupil produced by the application of belladonna, &c. to its vicinity. Dr. Paris alludes to another kind of sympathy, called by Mr. Hunter the *contiguous;*—it depends upon the continuity of parts; and an illustration is afforded by the relief given to internal inflammation by external fomentations or blistering.

The views of this accomplished physician with regard to the *modus operandi* of medicines are briefly as follow:

"The particular organs of the body may be excited into action through four distinct and different modes of communication.

"I. By the actual contact of the appropriate remedy. 1st. Conveyed by absorption, without decomposition; internally, *a*, through the branches of the thoracic duct, *b*, of the vena portarum; externally, *c*, through the branches of divided blood-vessels, *d*, through the branches of lymphatics. 2d. Conveyed by absorption with decomposition, by which one or more of its constituents are developed, and pass into the circulating current.

"II. By an impulse conveyed by the instrumentality of the nerves.

"III. By the sympathetic control excited by the stomach on distant parts.

"IV. By the operation of continuous sympathy, or of that which is excited by the mere proximity and continuity of parts."

Dr. Paris goes on to remark, that these are frequently antagonist operations, and that remedies are not to be regarded as similar agents, although they produce the same apparent effects, unless they act through the same medium. Indeed, he thinks it probable that no two remedies are exactly alike, and that "those medicines only are practically similar whose operations have been found, by experience, to continue similar under every condition of the human body; and which, moreover, owe such similarity to modes of operation which are compatible with each other, and consonant with the general indications of cure." The similarity of effect resulting from entirely different modes of operation, is well illustrated in the case of diarrhœa: this may frequently be checked by narcotics, astringents, or absorbents. An opiate, for instance, diminishes the increased discharge, by removing the irritation on which it depends; catechu might produce the same result, by restraining the flow of acrid fluids into the intestines; while chalk might act as efficaciously as either, by neutralising the acids, on the presence of which diarrhœa so frequently depends.

The observations of Dr. Paris on the *modus operandi* of the different classes of remedies are extremely interesting: our limits, however, oblige us to select but a few of these.—On the subject of vomiting, he is disposed to adopt the views of Dr. R. HARRISON. "It appears to me, (says this writer,*) that vomiting may be explained in the following manner:—the irritation of the stomach makes a call upon the brain for the aid of the diaphragm and the abdominal muscles, in order to expel its contents; the diaphragm then becomes contracted and fixed, the ribs drawn down, and the abdominal muscles drawn inwards, so that the stomach is pressed on all sides by voluntary muscles, which, together with its own contraction, expel the contents." From this it appears that, where the brain is much oppressed, it cannot transmit the requisite influence to the muscles; and so, disregarding the call from the stomach, vomiting is not excited. Under these circumstances, it frequently happens that the most powerful emetics fail in stimulating the stomach to evacuate its contents; an instructive instance of which was related by Mr. YEATMAN, of Frome, in a recent Number of this Journal;†—in

* *London Med. and Phys. Journal,* July 1819. † February, 1823.

his patient two scruples of sulphate of zinc, ten grains of sulphate of copper, in two doses, and twenty-four grains of tartrate of antimony, also in two doses, were exhibited, without producing emesis.

"Under such circumstances, venesection may in some cases prove a powerful adjuvant, by unloading the vessels of the brain, and thus restoring to the nervous system its necessary excitability. Where its powers have been paralysed by the operation of a narcotic, a copious draught of some vegetable acid, or the affusion of cold water upon the surface of the body, may impart efficiency to an emetic. The operation of nightshade, and some other narcotic poisons, may be adduced in farther illustration of this subject. An excessive dose of the Atropa belladonna produces symptoms of alarming stupor, and so difficult is it to evacuate the stomach under such circumstances, that as much as fourteen grains of tartarized antimony have been administered without effect: now if in such a case a copious draught of some vegetable acid be given, the emetic will be more likely to succeed. Here, then, we perceive that the brain, being paralysed by a narcotic poison, is unable to lend its aid to the muscles requisite for the operation of vomiting, until its energies are restored by the anti-narcotic powers of a vegetable acid. The practical precaution which this view of the subject affords is extremely important,—*not to allow the apparently inactive state of the stomach to induce us, inconsiderately, to augment the dose of an emetic:* for, although the stomach, for the reasons just stated, may be unable to void its contents by vomiting, it may nevertheless retain its sensibility, and be therefore liable to inflammation. Dr. Harrison has reported a case of this kind, where the practitioner, in attempting to excite emesis in an epileptic patient by a very large dose of sulphate of zinc, produced an inflammation in this viscus that terminated fatally."

As the activity of absorption is generally "in an inverse ratio to that of the circulation," it becomes of importance, in cases of poisoning, to shorten the period of nausea as much as possible, in order to prevent the absorption so likely to take place during this interval, before full vomiting is induced.

Diuretics produce their effects by very different modes of action, and there is no class of medicines more precarious in their operation. With the view of bringing together the principal facts upon this subject, Dr. Paris suggests their arrangement under three distinct classes:—1. Medicines which act *primarily* on the urinary organs; 2, medicines which act primarily on the absorbents, and *secondarily* on the kidneys; 3, medicines which act primarily on the stomach and prima viæ, and secondarily on the absorbents. Under the first of these classes are arranged the different saline preparations, as potass, soda, and nitre, which are carried to the kidney through the medium of the circulation, and may be detected in the urine. Let any person (says Dr. Paris) take several doses of nitre, taking care that the bowels are not disturbed by the medicine, and he will find, by dipping some paper into his urine, and afterwards drying it, that it will deflagrate, and indicate the presence of nitre." The substances enumerated undergo no change; but other salts are decomposed *in transitu,*—such are the acetate, citrate, and supertartrate of potass and soda. The vegetable diuretics have a similar mode of operation, as most of them contain a bitter principle, which is supposed to be separated by the digestive powers of the stomach.

"It particularly merits attention, that the diuretic operation of any body that acts by being absorbed is at once suspended if catharsis follows its administration, whether in consequence of the largeness of its dose, its increased solubility, or from the effect of its combination with some purgative; for it is a law, *that the processes of assimilation, and absorption from the duodenum, are arrested, or very imperfectly performed, during any alvine excitement.* The different effects of the saline compounds of the alkalies with tartaric acid, elucidate the truth of this law in a very striking manner: thus, supertartrate of potass, or cream of tartar, in well-regulated doses, acts, as we all know, upon the kidneys; the tartaric acid being, as I suppose in this case, abstracted and assimilated by the digestive process, and at the same time the alkaline base (potass) eliminated, and subsequently carried into the circulation; but if we increase the solubility of the compound, by reducing it to the state of a neutral tartrate (soluble tartar), or by combining it with boracic acid, or some body that has a similar effect; or, what is equivalent to it, if we so increase the dose of the cream of tartar that full catharsis follows its administration, then diuresis will not ensue, since no decomposition can take place under such circumstances; nor can it be carried by absorption into the circulation. Nitre, and those salts which are carried to the kidneys without previous decomposition *in transitu*, are subject to the same law; for, if we combine them with purgatives, their presence can no longer be recognised in the urine, as I have ascertained by experiment. Oil of turpentine, in doses of two fluid-drachms, may so excite the urinary organs as to produce even bloody urine; whereas a fluid-ounce will scarcely occasion any apparent influence upon those functions, because the increased dose acts upon the bowels, and consequently prevents its passage into the circulation.

"Sulphate of magnesia does not readily produce any diuresis, because it operates upon the bowels; but the experiments of Vitet and Bracy Clarke have shown that, if this saline compound be administered to the horse, whose bowels are not easily affected by purgatives, it acts powerfully upon the kidneys: and I will take occasion in this place to observe, that, on account of this inirritability of the bowels of the horse, diuretic medicines are more certain in their operation than in the human subject; a fact which, in itself, shows the importance of attending to the state of the bowels, during a course of these diuretics, which require to be absorbed before they can produce their specific effects.

"Equally necessary is it to attend to the state of the vessels of the skin; for if, during the administration of a diuretic, these vessels be excited by external warmth, its action may be diverted from the urinary organs to the exhalants on the surface, and occasion diaphoresis; but, if the surface of the body be kept cool, this diversion will not occur. So greatly, indeed, does this cooling the surface determine to the kidneys, that the usual diaphoretic medicines may, by an attention to this circumstance, be converted into powerful diuretics."

Mercury, in its various forms, is the only body ranked under the second class.

With respect to the third class, it is observed that a diuresis is frequently caused by substances acting on the stomach, which again sympathetically affects the other parts, especially in absorbents. Now this form of diuresis may be supposed to arise in various ways: the remedy may operate, *first,* " by diminishing the arterial action, and increasing that of absorption." It has been demonstrated (by Majendie) that the absorption of a poison is facilitated by depletion; and Dr. Blackall has proved the possibility of applying this truth to the cure of dropsy. It would appear, then, that means which control the circulation are capable of increasing the activity of absorption: " in this manner the digitalis purpurea acts as a sorbifacient; and it may be remarked, that it seldom, or never, produces its diuretic effects without a concomitant reduction of the frequency of the pulse: its power appears, too, only when it is administered in dropsy; in a state of health it will reduce the pulse, but not increase the discharge of urine." Again, remedies may act " by increasing the tone of the body in general, and that of the absorbent system in particular." Where dropsy is the consequence of debility, as after fevers, &c. any tonic, or even nourishing diet, may have a diuretic effect. The third manner in which this class of diuretics are supposed to act, is " by producing catharsis, and thereby increasing the action of the exhalants *directly,* and that of the absorbents *indirectly.* Hydragogue purgatives are of this kind: they excite a copious watery discharge from the bowels, and the absorbents are thus stimulated to supply the serous part of the blood, diminished in its due proportion. Such is the effect of elaterium. " In the whole circle of medicinal operations (observes Dr. Paris) there is nothing more wonderful than this, that an impression made on the internal surface of the primæ viæ by a few particles of matter should thus convey, by magic as it were, an impulse to the most remote extremities, rousing their absorbents to action."

The remarks of Dr. Paris on the other classes of medicines are of much interest, but our limits oblige us to content ourselves with having selected those which appeared to us of the most importance; besides which, some of the other interesting novelties, contained in the fifth edition of his work, have been pointed out in former Numbers of this Journal.*

Since these remarks were written, we have received " A Treatise on Materia Medica and Therapeutics," by Dr. Eberle, editor of the American Medical Recorder: of this we shall probably give some account in a future Number.

Various communications are to be found in recent Numbers of this Journal, tending to prove the efficacy of *iodine* in *bronchocele,* and some other glandular enlargements; and we deem the subject of sufficient importance to justify our laying before our readers some of the

* January and February.

most recent information relative to this powerful medicine, from the authority of BRERA* and BARON.† In doing this we shall quote freely from our respected contemporary, the Medico-Chirurgical Review. COINDET‡ imagined that the ill effects sometimes resulting from the internal exhibition of iodine might be obviated by its external application: this idea Brera regards as unfounded, and he believes, further, that its internal administration is equally safe, and more efficacious, than the other, except under particular circumstances. He uses the following formulæ:

"1. *Tincture of Iodine.* Made by dissolving 48 grs. of pure iodine in an ounce of alcohol (at 35). This is the preparation most frequently used at first by Dr. Coindet, who, as well as Brera, recommends it being used *fresh,* as it is liable to decomposition in a few days. The dose is from five to twenty drops for adults, three times a-day. Twenty drops contain about one grain of iodine.

"2. *Pills of Iodine.* Made by forming one grain of iodine into two pills, with elder-rob and liquorice powder; one to be taken morning and evening.

"3. *Iodine Ointment.* Made by rubbing up a dram of pure iodine with an ounce of lard, or half a dram of hydriodate of potass with an ounce and a half of lard; the former in the quantity of a scruple, the latter about the size of a filbert, rubbed on the part.

"4. *Solution of Hydriodate of Potass.* This preparation is stated to be preferable to any of the foregoing, producing their good effects without their inconveniences. It is formed by dissolving 36 grains of the hydriodate in an ounce of distilled water, and is given in the same dose as the tincture.

"5. *Solution of the Ioduretted Hydriodate of Potass.* Formed by dissolving 36 grains of the hydriodate and ten grains of pure iodine in ten drams of water. This is said to be a still more efficacious preparation than the preceding, and requires to be given in small doses,—viz. five or six drops, three times a-day, to begin with."

Its effects upon the human frame are thus described:

"When iodine is cautiously and gradually introduced into the system, it affects it in a general manner, analogous to that of mercury, but very different in the consequences. The first, and what may be called the *salutary* effects of iodine, are an increase of appetite and of the strength of the pulse: whenever these are produced, we must watch with the greatest care that these salutary limits are not exceeded, and the pernicious consequences of an over-saturation of the system induced. The complete impregnation of the system is indicated by the change of the above-mentioned increased action of the pulse into decided frequency and quickness,—by a sense of heat and irritation of the fauces,—pain of the orbits or eye-bills, with obscured vision,—pain of the internal

---

* *Saggio Clinico sull'Iodio, e sulle differenti sue Combinazioni e Preparazioni, &c.* Padua, 1822.

† *Illustrations of the Enquiry respecting Tuberculous Diseases.* By JOHN BARON, M.D. 1822.

‡ *Observations on the remarkable Effects of Iodine in Bronchocele and Scrofula, &c. &c.* Translated by J. R. JOHNSON, M.D. 1822.

ears and gums, with occasional salivation,—head-ache, restlessness, loss of sleep, with swelling and pain of the diseased organs, (*e. g.* thyroid and other glands,)—and an increase of appetite, sometimes to a degree of voracity. In some persons the submaxillary glands become painful and swollen; and a similar state of the mammæ, *with eventual diminution of their natural volume*, takes place in some females. When given from the first in an over-dose, iodine produces a strong burning sensation in the fauces, which frequently extends down the œsophagus to the stomach and whole intestinal canal. In a still higher degree of saturation (or *iodization*, as the author calls it,) of the system, to the above-mentioned symptoms succeed very considerable emaciation, even in the space of a few days,—excruciating pains in the orbits and eyes, with great defect of vision,—and similar pains in the diseased parts; the strength vanishes; neuralgic pains are experienced in the stomach, chest, bowels, &c.; the sleep entirely fails; and there is obstinate palpitation of the heart, with tremors, convulsions, or palsy of the extremities. To the excessive appetite succeeds complete anorexia; and the factitious disease finally terminates life, in a short time, by universal inflammation of the nervous and vascular systems, *(profonde angioitidi e neuritidi.)*

"Upon the appearance of the milder class of symptoms above mentioned, the immediate suspension of the medicine (which ought always to be done,) sometimes is found sufficient to put a stop to them in a few days. For allaying these deleterious effects, rigorous regimen, copious mucilaginous drinks, and the tepid bath, are recommended; and where the topical affection of the goitre, or other tumors, runs high, fomentations, poultices, leeches, &c. are prescribed; and general bleeding is advised where there exists a high phlogistic state of the whole system.

"As these symptoms sometimes show themselves all at once, we ought to be cautious in not too hastily increasing the dose in cases wherein no obvious effects are produced. After the bad symptoms are allayed, the medicine is to be repeated with the same precautions as in the case of mercury and arsenic."

According to Brera, it gives rise to particular action, not only in the thyroid gland, but in the uterus,—increasing scanty, and checking profuse menstruation; sometimes likewise diminishing the size of the mammæ: and hence he is led to hope it may prove useful in organic diseases of the uterine system.

Dr. Baron is of opinion that we have no medicine possessed of powers equal to those of iodine, in the removal of morbid growths by promoting their absorption. His attention was first drawn to the subject by the report of Dr. Coindet, and he has since tried it in a variety of cases different from those mentioned by that gentleman.

"The first I shall mention is one of physconia hydatidosa. It much resembled a case which I have described at page 95 of my Enquiry. The abdomen was as large as that of a woman in the last stage of pregnancy. The tumor had been more than once reduced in size, by the long continual use of mercury and liquor potassæ; but it never was effectually removed. More than once its bulk was very much diminished, by an event which establishes its original character, and justifies the name which I have assigned to it; I mean the disruption of one or more of its cysts, and the discharge of the contents into the alimentary canal; such fluids as hydatids are known to contain in various stages of their progress, having, after the events just described, been discharged both from the stomach and per anum.

"This patient began the use of the hydriodate of potass in solution, on the 6th of October, 1821. She took at first eight drops twice a-day, and continued them very regularly till the 23d of March, 1822. By this time a marked effect had been produced on the size of the tumor; but, in consequence of some unpleasant feelings about the stomach and head, the drops were discontinued, and not resumed till June the 22d.

"From the use of this medicine, a very striking absorption of the diseased structure has taken place. Before she began it, the bulk was nearly as great as at any former period: now, it is not discernible by the eye; and it requires a pretty accurate examination by the touch to discover the remains of the substance, as she calls it, in the left iliac region."

The hydriodate of potass is the form to which Dr. Baron appears to give the preference; from which he informs us that he has scarcely found any of those inconveniences mentioned by Dr. Coindet: his experience likewise agrees with that of Professor Brera, "that mere friction or inunction will not, in many cases, be successful without also giving it internally.

Narcotics, in the form of vapour or fumigation, have lately been recommended by HUFELAND in the cure of *epilepsy*. The hyoscyamus and belladonna were selected, and opium occasionally added to increase their effect; the method of their exhibition being as follows:—Six ounces of the former, and ten or twenty grains of the latter, were moistened with water, and spread on an iron spatula, to which heat was applied by a spirit-lamp, until carbonization of these substances took place, and a vapour-bath filled with the fumes. The patient was submitted to the action of the atmosphere thus charged for a quarter of an hour, or rather longer; the result of which was some increased perspiration, and a tendency to congestion about the head: sometimes the more unpleasant symptoms of vertigo, tremors, or spasms occurred, requiring the active superintendance of the physician, and variations in the quantity of the narcotics corresponding to the circumstances.*

### SURGERY.

Whilst the physician and physiologist have to lament the occasional failure of their best endeavours for the advancement of their respective branches of science, and cannot but admit that they too frequently have been walking in a circle, the surgeon's progress, if not rapid, is at least constant, and every step he takes leads him nearer to perfection,—that is, to the perfection of his *art*. The last six months, if it be not distinguished by any very brilliant works on surgical subjects, has been fertile in recorded operations of great importance,—in some few novel-

* *Nouveau Journal de Medecine,* October 1822.

ties,—and in many highly interesting observations, tending to establish and confirm particular points of practice, hitherto subject to doubt and uncertainty. We shall first detail what has been done towards the advancement of the operative part of our art; we shall then endeavour to appreciate what we owe to those authors who have published on this branch of science during the last half year; make some incidental remarks upon the present state of practice in some of those diseases usually denominated surgical; and enumerate (for we can do little more without the assistance of engravings,) one or two new instruments that have been presented to the profession during the period under consideration.

In the first rank of important operations we have to record the *ligature of the arteria innominata* by Graefe, and also by Dr. Mott, of New York; and of the *subclavian artery* in no less than three instances, two in this country and one in St. Petersburgh. All these cases, excepting the last, of which we only know the fact that the patient did recover, but are not in possession of the details, proved fatal; but in the first operation, that of the ligature of the arteria innominata, the patient survived sixty-eight days, and died of diseased lungs. The subject of this operation was a sailor, about thirty years of age, who was affected with an aneurism of the right subclavian artery. During the operation, the patient was put upon his back on a table, in such a manner that his head was hanging down on one side of the table, and the arteria innominata drawn up a little above the manubrium of the sternum. The professor then made a longitudinal incision, near the anterior edge of the sterno-mastoid muscle, down to the sternum, exposed the carotid artery, and followed its course till where it unites with the subclavian artery to form the trunk of the arteria innominata. This was effected with very little difficulty, and the arteria innominata became distinctly exposed to sight. By means of a needle, curved in a particular manner for that purpose, a ligature was then passed round the arteria innominata, and both ends of it tied together. No alarming symptom ensued, and the ligature, which was considerably broad, came away about a fortnight after the operation, carrying out just that portion of the artery where the innominata is divided into the carotid and subclavian artery. Some time afterwards, however, a hemorrhage suddenly arose, which, though considerable, was soon stopped by the application of cold water and pressure. The patient then complained of pains in the tumor formed by the aneurism, in which fluctuation was distinctly to be felt; and this determined Dr. Gräefe to make an incision into the aneurismal sac. A considerable quantity of pus and grumous blood was thus evacuated; and matter continued to be discharged daily from this opening, whilst the other was filled with granulations, and went on exceedingly well. The patient, however, got into a state of fever, began to spit blood, and to throw up matter; and so he died on the sixty-eighth day after the operation had been performed.—On dissection, the lungs were found in a diseased state; and in the arteria innominata a clot had been formed, extending from its origin to where the ligature had been applied. By an injection made into the aorta, the arteries of the right arm and right side of the head were entirely filled; the circulation in these parts having been fully re-established by anastomosing branches.

We have described the operation performed by Mr. BRODIE in a former Number of this Journal: in this case the aneurism, situated in the axilla, was small, had not existed any great length of time, and the patient was about sixty years of age. The result was fatal on the sixth day. In Mr. TRAVERS's case, the patient was about the same period of life; but the tumor was large, insomuch that the parts were greatly thrown out of their natural situation, rendering the operation difficult and tedious: it occupied nearly two hours. The result was unfortunate, the man dying on the third day.

Dr. MOTT's case of ligature of the arteria innominata is related in a German periodical work,* and the leading facts are these: The patient was a sailor, fifty-seven years of age; the length of time that the aneurism had existed does not appear, neither is its extent or situation very accurately described; the man was affected with a violent cough, and is said to have been very feeble. On the 11th of May the operation was performed, in the following manner:

"Two incisions were made, one in the direction of the clavicle, and the other along the sterno-cleido-mastoideus. The carotid was laid bare, and traced towards the subclavian, which was found so diseased that they had no alternative but to tie the innominata. They accordingly carried the incisions deeper, and, separating the recurrent and the phrenic nerves, they came to the division, and passed the ligature, with a curved needle, about half an inch higher. The parts were then brought together by suture, and the wound bandaged. There were only three arteries divided,—a branch of the internal mammary, and two branches severally from the inferior and superior thyroid. He lost only about three ounces of blood. The whole operation lasted about an hour.

"The patient immediately after felt quite well; pulse 69; temperature of the arm nearly the same as the other; respiration unchanged. From this period to the twenty-second day after the operation, he continued to improve; the suppuration went on well, the ligatures came away without accident, and the pulse, which had at one time risen to 120, was reduced by venesection to its natural standard; the cough was disappearing, cicatrization was going on properly, and the swelling becoming gradually less. He was in high spirits, and was so far recovered that he walked daily in the garden of the hospital. All on a sudden, however, on the twenty-fourth day, a hemorrhage from the wound took place; and though it was soon got under, and there was little loss of blood, it recurred twice in the next two days, respiration became painful, and the patient died on the twenty-sixth day.

"Eighteen hours after death, the wound was black and fœtid. There was no trace of inflammation in the arch of the aorta, the origin of the arteria innominata, or in the lungs. The internal membrane of the innominata was smooth and soft, and its parietes were so thick that there was only room for a crow-quill to pass. The subclavian artery opened

* LANGENBECK's *Neue Bibliothek*.

into the tumor; the carotid was filled with coagulated blood. The arteries of the arm were healthy. The clavicle was carious, and almost separated in the middle. The death was evidently caused by extensive suppuration."

Instances of the successful result of the *ligature of the external carotid and iliac arteries,* are now so multiplied as to have lost much of their interest. Two cases of this kind are related by Dr. ARVEND, of St. Petersburgh.* Both patients were about forty-four years of age, and the ligatures came away on the sixteenth and seventeenth days respectively.

In England, surgeons appear to be generally decided as to the propriety of suffering the ligature to fall off spontaneously in the operation for aneurism, and we are happy to find this, which we consider sound, practice has found an advocate in Professor VACCA, whose experiments and arguments appear to us to be conclusive on this subject.† Two cases where SCARPA's plan was successfully employed are, however, detailed since our last Retrospect. They were operations for the cure of popliteal aneurism: in both, the patients were in the prime of life, and the ligatures were removed on the fourth day. We have so recently stated our objections to this method of performing the operation, that we do not consider it necessary to renew the discussion now: we must not, however, omit noticing in this place a late addition of M. Scarpa to his usual apparatus,‡ for it seems that he still adheres to his plan of removing the ligature at the end of the third day; and all his contrivances have one object in view, that of rendering this second operation (for such in fact it is) as little painful as possible to the patient. This new plan consists in passing a hollow instrument down to the knot of the ligature of the vessel, by means of that portion of it which is left out of the wound. This sound has a groove throughout its whole extent, and in which a very small knife is to be passed down to the knot, which is in this way to be cut, or rather *sawn*§ asunder: the blade of this knife is to be only five lines in length, and sufficiently fine to pass readily into the groove of the sound. This is the outline of M. Scarpa's proposition. English surgeons, we believe, are not likely to be induced to resort to all these unnecessary and complicated means to do that which we conceive it to be much wiser and safer to leave undone.

We shall subjoin one passage from M. Scarpa's letter, in order to show that, even with the additional facility afforded by this new contrivance, some little manœuvring is necessary, and a good deal of irritation must be produced by taking away the ligature, even after the knot is divided. "The hand of the operator (he says) feels the division of the ligature: nevertheless, on the third day after the operation, as all foreign bodies remaining in the wound are glued together by the elastic substance which is thrown out, and as it is likewise important to be assured that the ligature is completely divided, before the sound is

* *Salzburger Zeitung Med. und Chir.*

† *London Medical and Physical Journal*, May.

‡ *Revue Medicale*, Mai 1823.

§ "Un petit mouvement de scie," is recommended to be given to the instrument.

withdrawn from the bottom of the wound, in order to prevent the artery from being pulled or shaken, it is essential to extract from the wound immediately the inner cylinder, by means of the waxed thread attached to it. The sound is afterwards drawn out with care, and with it the ligature comes away without difficulty."*

Two cases of *Cæsarean section* are recorded in this Journal; one of which was successful. We have certainly nothing to encourage us to perform this operation in our own country; but it would appear that climate, mode of living and diet, or some other unexplained causes, do subtract considerably from the peril of this most formidable operation; or, at least, that we meet with a greater number of recorded instances of its successful result among foreigners in one year, than we can boast of possessing from the earliest records to the present time. We confess that we have strong objections,—we had almost said prejudices,—against the performance of this operation, but which certainly do not apply to that which follows.

M. BORRONE has related an operation of the same kind, performed upon a female aged thirty-six, who died of dysentery, at the full period of gestation. He extracted from the body, twelve minutes after death, a small emaciated female child. It was some time before the infant uttered the least cry, but it felt warm, breathed, and the pulsations of the heart were manifest. Thirty-two minutes after extraction, it began to suck. It gradually acquired strength, and, towards the middle of the year 1822, was tolerably robust.†

An interesting case of *bronchotomy* has been published by Mr. JAMESON, of Baltimore. The subject, a boy aged between four and five years, was visited by that gentleman, in consequence of a water-melon seed having descended into his windpipe about seven days previously.

"The child was in a high fever, with incessant croupy cough. I requested the advantage of the opinions of Drs. Jennings and Cromwell.

"The child was bled, took several emetics, which had the effect of affording the most decided benefit. The vomitings, by bringing up great quantities of phlegm, relieved his cough and respiration so much, as to encourage his parents to hope that this plan would ultimately succeed in relieving him altogether. For three or four days he would be free from almost any appearance of disease; then he would be threatened with strangulation, and severe and incessant cough would be excited, and continue till he was quite exhausted. Thus he continued, upwards of three weeks after I first saw him, to change from a state of extreme danger to one of apparent health, except his gradual loss of strength and flesh. After this period the vomits began to lose their beneficial effects, and, ultimately, evidently did him harm. The parents having resisted our advice respecting an operation, I gave them expressly to understand that, unless they consented to it, I would no longer be

* Lettre de M. SCARPA, Mars 1823.

† *Medical Repository*, January.

responsible; nor would I be willing to perform an operation when no hope was left of saving him by it. I requested a final consultation with the gentlemen above named.

"On the 31st of August we met, and agreed that, as there was no chance of relieving him without an operation, if the symptoms should not again remit and leave us a reasonable hope, which his situation on that day did not, that then the operation should either be promptly performed, or all idea of it abandoned for ever.

"On the 2d day of September, one month after my first visit, and five weeks after the accident, I engaged in the operation, in the presence of Drs. Cromwell, Jennings, Wright, Dickson, and others. I made an incision about two inches in length, parallel with and in front of the trachea, terminating below near the sternum, and above about the middle of the thyroid cartilage. The integuments were much thicker than I expected: that part of the wound over the *rings* to be divided was more than half an inch. Having completed my incision, I passed the point of the scalpel between the thyroid and cricoid cartilages, and cut downwards so as to form a wound through the rings of the trachea, of about three-fourths of an inch. Here it may be proper to notice some change of my views growing out of the circumstances present. I had provided myself with delicate forceps, formed out of silver wire, hoping, if I could not take hold with the forceps, that, by turning the seed across the tube, I should enable the organs of respiration to throw it out of the wound or through the glottis; and I was not entirely at ease about a risk which I imagined there was of the seed being forcibly lodged in the glottis, and giving us some trouble.

"The vessels divided by the knife bled so freely as to induce me to hope that I could derive advantage from this occurrence. I determined to open the wound for a short period,. so as to admit blood into the trachea, with a view of forming coagula about the seed, or to stop up the trachea as much as possible, with a view of obtaining a more complete expelling power from the respiratory organs. Finding that my forceps were, though small, still too large for a space so confined as that deep between the sternum and the chin of a child, I resolved to trust to moving the seed, and irritating the lungs with a common probe, believing the coagula would materially facilitate my design. I was not aware that my probe had been taken out of my case, and there being none at hand, I passed down the forceps with their chops pressed together. The moment I touched the seed, and, no doubt, turned its flat side across the tube, which was somewhat choked with coagulated blood, it was thrown through the glottis as out of a pop gun, in consequence of the irritation excited by touching the inner surface of the trachea at its bifurcation.

"It was instantly perceived that the child was relieved of much of his sufferings; and so sensible was he of what had been done for his relief, that he lay perfectly quiet, and bore the introduction of three sutures without a struggle or complaint. A slight symptomatic fever followed the operation; but there was very little cough, and that was free from the peculiar croupy sound during the presence of the seed. The fever yielded to two or three mild cathartics. At this time (19th

September,) the child is in fine health, but a slight sore remains at the wound, in consequence of the sutures having given way before the skin had united; but the trachea closed up perfectly after the first afternoon, at which time a little air passed through the wound."

Mr. Wishart has favoured us with an account of a case of *fungous hæmatodes of the eye-ball*, and which was successfully extirpated. The subject of this operation was a boy nine years of age. "On examination, the eye was observed to have a general turbid appearance; it was devoid of lustre; and, on minute inspection, the cornea was found to be transparent, but numerous vessels passing over the sclerotica into it. The pupil had a slightly serrated appearance; was moderately dilated, but did not change its size on variations being made in the degree of light. In the posterior chamber an opacity was observed, resembling a yellow dusky membrane, lining the whole posterior part of the eyeball, more distinctly perceived when the eye was viewed laterally. The vision was nearly gone; the eye watered profusely, especially when exposed to the light, which occasioned considerable pain and irritation; had at times a shooting pain in the frontal edge of the orbit, of short duration; pulse natural; general health good.

"About two months ago, received a blow on the eye while at school. The afternoon of that day it gave him no uneasiness, but next morning he felt great pain in the eye, and the vision was almost entirely lost. The effects of this injury were apparently removed by free local bleeding and antiphlogistic treatment, under the care of Mr. Stewart, of Queensferry; and in a few weeks he was able to return to school. About ten days ago, the eye again became inflamed and painful. Six leeches were ordered to be applied to the forehead, and a dose of infusion of senna to be given in the morning."

It is needless to detail the treatment employed during the space between the 18th of May and the date of the operation, since it had no influence over the disease. On the 3d of July, the constitution began to sympathise with the local malady; febrile symptoms supervened, with great pain in the eye and frontal margin of the orbit.

"In consultation with Mr. Gillespie, it was agreed that the removal of the eyeball was now expedient; but, with the view of lowering his system, two cupfuls of blood were directed to be taken from his arm on the 4th. On the 5th, he had a dose of salts; on the 6th, six or eight leeches were applied to the forehead and temples; and on the 8th, the salts were to be repeated in a full dose.

"9th July.—The inflammatory symptoms having greatly subsided, and the disease in the internal part of the eye being still on the increase, the iris being now in close contact with the cornea, and the pupil completely closed by the opaque matter, it was resolved to extirpate the eyeball; and the operation was performed in the following manner:

"The temporal angle of the eyelids was divided with a scalpel, which was then passed round, first at the frontal aspect of the eyeball, from the external to the internal angle, and the muscular and other attachments divided; the lower segment was divided in a similar manner; and the fore-finger of the left hand being passed from the nasal angle backwards, the optic nerve was found to form the only remaining connexion; it was readily divided with the same scalpel, and the whole removed from the orbit. The lacrymal gland was dissected out; the vessels were allowed to bleed for a few minutes. The divided eyelid was then brought together by a single small ligature; and, the clotted blood being removed, two small strips of dry caddis were pushed gently into the orbit between the eyelids. Over this a pledget of simple ointment, secured by a compress of caddis, and a few turns of a double-headed roller passed round the head. The little patient was put to bed. He bore the operation uncommonly well: it occupied only four minutes.

"After being in bed for about an hour, a slight dropping of blood was observed; but, as he had fallen asleep, it was not thought necessary to remove the dressings, and it did not increase.

"11th.—Slept well both last night and the preceding; complains merely of the stiffness of the dressings. Pulse natural: a great part of the dressings removed; low diet.

"13th.—Complained of slight pain of the orbit this morning, of very short duration: dressings entirely removed; suppuration begun; parts look well; became very faint during the dressing, apparently from fear and change of posture, it being necessary to take him out of bed for the convenience of a proper light.

"15th.—Dressings changed daily; discharge very moderate; not the smallest derangement of functions, and no complaint, except of hunger.

"17th.—Has been out of bed all day, and walking about the drawing-room; discharge healthy; allowed more nourishing diet.

"21st.—I found the patient so well, that I did not find it necessary to visit him again; and I heard no more of him till the beginning of March of the present year, when he called to show me a small tumor which had been observed in the orbit. On examination, I was happy to find that it was merely a granulation projecting a little, with a narrow neck: on lifting it up with the point of a probe, it bled a little; and the little fellow, being evidently much alarmed, dropped off the chair in a state of syncope. I was unwilling, on this account, to do any thing more, but requested him to return in a day or two, intending to cut off the projection with the curved scissors, or put a thread round it, as might appear most advisable. However, on the following day his father wrote to me, that 'the bit of flesh had been so disengaged by you yesterday, that it came out when his mother was washing the eye. The place is a little red, but he complains of no uneasiness.'

"Being anxious to ascertain whether this cure continued permanent, I wrote to Mr. L. in July, and received the following very satisfactory answer: 'I am happy to say that, since you last saw my son, the eye has appeared to be quite healthy and well. He has not, upon any occasion, complained of the slightest uneasiness in it, or of any affection of the head; and his general health has been perfectly good. It is consoling to think that the consequences of the operation have hitherto been, and I now sincerely hope will continue to be, so favourable and satisfactory to all concerned.'"

On the dissection of the eyeball, the appearances presented were precisely the same as those so accurately delineated by Mr. Wardrop. The origin of the disease in the retina was finely and satisfactorily illustrated. The optic nerve was quite healthy; the sclerotic and choroid coats were of natural texture; the cornea was a little softer than natural, and not perfectly transparent; the lens was pushed into contact with it, and seemed smaller than usual, and flattened. The diseased mass into which the retina had been converted, connected only to the optic nerve, floated loosely in various folds, occupying both chambers of the eye. The eyeball did not appear to be at all enlarged.

This case we consider as valuable on several accounts: it is nearly, if not quite, solitary as an instance of success in this deplorable malady; the length of time that has elapsed since the child's recovery (a year and a half) affords reasonable ground for hoping that the cure will be permanent; and it teaches us the propriety, or rather the necessity, of operating at an early period of the complaint, and the importance of doing so previous to the disease having attacked the optic nerve.

Dr. DRAPES has published a case of *sarcocele*, in which the testicle was removed; and we are induced to mention it, because, from the peculiar circumstances of the operation, the spermatic artery was not taken up separately, but the whole cord was included in the ligature. This practice has been represented by many writers of eminence as fraught with danger, whilst others of equal celebrity recommend it as the preferable method. It is of importance to come to some decision upon this practical point, as the ligature of the whole cord, if it be equally safe, certainly simplifies and shortens the operation very materially. Mr. PEARSON,* in speaking upon this subject, recommends the ligature of the whole cord, and observes that the knot should be drawn *very tight*; in which case all the evils usually ascribed to the inclusion of the vas deferens are averted. In the case to which we now allude, it appears that much distress was felt by the patient in the groin on the day following the operation, and that the constitution was also very much disturbed: two full bleedings, however, and a purgative, produced a speedy alleviation of all the unpleasant symptoms, and they did not recur, the case going on to recovery in a very satisfactory manner. We can safely say, in addition, that we have never known any dangerous or alarming symptoms to arise from the ligature of the whole cord, and that we are therefore decidedly friendly to that mode of operating.

M. DUPUYTREN† has lately recommended and practised a mode of curing *prolapsus ani*, which he considers as novel: it consists in cutting off a greater or less number of the cutaneous and projecting folds of the verge of the anus. The operation contracts the opening by drawing it together, almost in the same manner as a purse when the strings are drawn tight. Ten or twelve patients have been operated upon in this way, and have been cured, without any unpleasant symptoms. No dressing is required; attention to cleanliness being alone sufficient to

---

* *Practical Observations on Cancer.*

† *Journal Universel des Sciences Medicales*, Octobre 1822.

produce complete cicatrization in twelve or fifteen days. We have mentioned this operation, although we do not see in what it differs from the plan recommended by Mr. HEY, of Leeds.* We should, for our own parts, be inclined to adopt it only in extreme cases, since we do not think so lightly of operations performed about the anus as our neighbours: we are convinced that, in many instances, they are attended with considerable risk, and produce most formidable constitutional disturbance; and it is, therefore, only in those instances in which the evil is great, and life is rendered burthensome by the extent of disease, that we should urge or press upon the patient a mode of relief which we consider as by no means free from danger.

On the subject of *lithotomy*, we have one or two novelties to record: the first is a new method of opening into the female bladder, proposed by M. LISFRANC.† To this operation we have not any thing to object, but we are not aware of its having been performed in this country. Cases of calculi in the female are not very frequently occurring, and they most commonly admit of extraction without the necessity of having recourse to cutting instruments. The forceps invented by Mr. WEISS have been used upon several occasions with success, since Sir ASTLEY COOPER published his cases in the Medico-Chirurgical Transactions.‡ Experience fully warrants our former approval of this ingenious invention; and it, at the same time, tends to confirm us in the belief that the cautious and gradual dilatation of the urethra is, on every account, to be preferred to the completion of the whole process at one time: a question which Sir Astley was anxious to have decided by the only infallible test—that of experience.

M. ARNUSAT§ has invented an instrument for the purpose of breaking down calculi in the male bladder, and which are afterwards voided in their pulverised state. The instrument consists of a pair of pincers, concealed within a sound. No experiments have, however, yet been made with this instrument, excepting on the dead body. While we acknowledge the ingenuity of M. Arnusat, we cannot bestow our unqualified approbation upon this invention, or rather upon this application of an invention: in the first place, it can only be available in cases of soft and friable calculi, and cannot be supposed capable of grasping stones of any great size. These inconveniences would, of course, restrict its use in a very great degree; but we should apprehend that, in the living subject, breaking down a stone would be but dangerous practice, unless we were quite sure of reducing the calculus to such a degree of minuteness as to insure the evacuation of all the fragments; otherwise, we should probably be only leaving the nuclei of future stones, and multiplying the evil indefinitely.||

* HEY's *Surgical Observations.*

† *Revue Medicale*, Janvier.—See our last Number, p. 440

‡ Vol. xii. § *Bibliothèque Universelle.*

|| Instruments for this purpose are no novelties. We find one described by M. LEROY, the principle of which consists in sawing the calculi within the bladder. Mr. ELDERTON published an account of an instrument, in the year 1819, for destroying stones within the bladder, &c. &c. All the experiments were made, however on the dead body in both instances.

A much more judicious and scientific application of a similar instrument has been made in this country by Sir Astley Cooper, unless we are much misinformed, and in which he was fortunate enough to *extract* from the bladder of a clergyman several small calculi, by means of an instrument of Mr. Weiss's construction.

We have still one more novelty, having reference to the operation of lithotomy, to announce: it is the proposal of M. CAMPANA* to take hold of the calculus, for the purpose of extraction, by its largest diameter. We have so recently given† M. Campana's reasons for this practice, that we need not repeat them here. We shall offer no comment upon them, but merely say we think his plan, at least, deserving of consideration.

In a late Number of a foreign publication,‡ we find a letter from Mr. SANSON, relating the *recto-vesical operation.* It is in answer to a Mr. Fortis, who, in a letter to M. Riberi, has thrown much undeserved odium upon this operation, and misrepresented the result of the case in which it had been successfully performed by M. Sanson. From this gentleman's statement it appears that M. DUPUYTREN operated in this manner upon a lad, thirteen years of age, about the latter part of the year 1821; that the operation was tedious and difficult, the stone being of an enormous size, (two inches four lines in length, and one inch eight lines in breadth;) and the patient died in consequence of the formation of an enormous abscess on the thirty-sixth day. The size of the stone, and the difficulty of extracting it, in consequence of the incision having been made at first too small, and subsequently enlarged,—the forceps being found too slight to embrace and retain the stone,—all contributed to produce such a degree of irritation as to lead to the unfortunate termination of this case. It is added that M. Dupuytren has not since repeated this operation. The case operated upon by M. Sanson recovered perfectly.

Dr. PHYSICK§ has recorded a case of *ununited fracture of the lower jaw*, treated successfully by passing a seton through it. Our trans-Atlantic brethren have now given us several instances of the same kind, the result of which has been equally fortunate: with us, however, these operations have not in general succeeded.

Several detached cases of operations, successfully performed, are related in the various periodical works during the last half year; but, as there is nothing novel or instructive in the mode of performance, nor any particular difficulties to be overcome in the progress of the cure, we shall not waste the time of our readers by detailing them. One very remarkable story,‖ however, we must mention, which is the extraction of a single fork from the stomach of a man, by M. RENAUD, a surgeon at Romans, (department of the Drôme.) The account of

---

* *Annali Universale de Chimie.*

† *London Med. and Phys. Journal*, June.

‡ *Journal Complémentaire*, Mars.

§ *Philadelphia Journal*, No. 9.

‖ *Tablettes Universelles.*

this case, however, is very imperfect; inasmuch as we are merely told that the young man was considered out of danger, and *expected* to recover in a short time. The fork was eight inches long, and produced the most alarming symptoms.

We find, in an Italian Journal,* a case recorded in which the *uterus* was extirpated from its natural situation by Dr. SAUTER. This appalling operation was undertaken at the pressing desire of the patient, suffering under the agonies of a cancerous affection of that organ, for which she had been under treatment from the middle of October 1821, to the 28th of January, 1822, the day of the operation. The woman was fifty years of age; she had borne six children, and had ceased to menstruate about four years. Having tried in vain to bring down the uterus with the index-finger of the left hand, M. Sauter introduced that and the middle-finger to the extremity of the vagina, and, with a straight knife, he separated it from the uterus, cutting the termination of the vagina little by little between his two fingers. In operating in this way, his object was to draw the uterus downwards, to detach it afterwards from the cellular membrane surrounding it, either with the handle of the knife or with the fore-finger of the right hand. He found this could not be done in any way that he made the attempt; but, having advanced so far, it was necessary, he thought, to proceed, believing that the cancerous ichor would infect the cut surfaces. Re-examining the parts, he found the bladder had been wounded in separating it from the uterus, and he therefore determined to extirpate this viscus. He accordingly introduced two fingers of his left hand into the wound, between the bladder and uterus, and, cutting little by little between the fingers, he divided all the adhesions, until, with the finger which followed the borders of the uterus, he could penetrate into the *cavity of the abdomen;* he then passed the fingers of his left hand as high as possible to the lateral ligaments, seized and divided them as near as he could to the womb; he then separated it from the ovaria, from the fallopian tubes, and the other ligaments. He then laid hold of the fundus with four fingers, and tried to invert it, but in vain, as the action of the abdominal muscles *pushed down the intestines.* Having desired the patient to restrain this action, and the intestines being pressed upwards by an assistant, he inverted the womb, and drew it out between the labia; the excision was then easy. This horrible operation lasted three-quarters of an hour: only one pound and a half of blood was lost; the woman, however, fainted towards the termination. The *intestines being put back into their place*, the vagina was filled with charpie. We need not be surprised that this poor woman was troubled for several days with vomiting, and that her belly was painful; but in a short time all these symptoms disappeared, and, excepting that some urine escaped from the wound, her recovery went on regularly. Suffice it here to say that, after a period of about three months and a half, the patient quitted the hospital. She, however, only remained at home seven days, and expired about a fortnight after her re-admission. M.

* *Annali Univ. di Medicina,* 1823.

Sauter describes this operation as not being either very painful or accompanied by much hemorrhage; but is of opinion that wounding the bladder is inevitable.—We shall offer no comment on this case!

Mr. CHURCHILL has presented us* with some additional cases of the successful employment of *acupuncturation*, and promises us soon "a body of evidence," which shall dissipate the most obstinate scepticism. For our own parts, we are not at all sceptical upon this subject: we are fully sensible that the operation has been followed by immediate and permanent relief in many instances, particularly in that of a nobleman of high rank in the county of Sussex, and who has contributed very largely to extend its reputation, and to enlarge the sphere of its practice; but we are also aware of its having been employed in vain in fully as many cases, and that in others the benefit has only been temporary. We do not at all intend to depreciate the utility of this simple and easy remedy by this statement, but we merely wish to put our brethren in possession of the *per-contra side* of the account,—lest, if they should meet with a disappointment upon the first occasion of their applying the needles, they may hastily and rashly abandon the practice, as supposing it to be founded in delusion. Mr. Churchill very judiciously appears anxious to restrict the utility of this operation to the relief of that painful disease for which it is more particularly recommended. We shall only add, that the cases he has published are detailed in a very perspicuous, and apparently a very candid manner, and are perfectly satisfactory to our minds.

We have an account of a new operation for *artificial pupil*, by Dr. WELLER, of Dresden,† which will, perhaps, be best understood from the detail of the case in which it was performed.

"Miss L—, seventeen years old, was, in consequence of a probably scrophulo-rheumatic ophthalmia, blind of both eyes, and had been so five years, yet there remained in her right eye a feeble sensibility to light. From all circumstances, it was concluded that the inflammation had affected the whole globe. The cornea was transparent, the anterior chamber tolerably large, but the bulb somewhat enlarged, and around the cornea was a bright bluish ring in the upper part of the bulb. Schlerotic staphyloma had taken place, along with varicose vessels; the iris was spongy, and the pupil closed from firm exuded lymph, and drawn tunnel-formed backwards; and the ciliary margin, here and there, adherent to the cornea. In this eye, according to our author, no one could perform iridodialysis, because the iris, on account of the firm adhesion, would not have admitted of separation, and the instrument must have torn the iris. He proceeded, therefore, in the following manner:

"He pierced the cornea with a cataract-knife half a line from its circumference, towards the outer angle of the eye; introduced through this opening a hooked needle, which was not larger than the cataract-

---

* *London Med. Repository*, May.

† *Quarterly Journal of Foreign Medicine, &c.* April.

hook of Beer, but bent in a larger circle, therefore more of a needle-shape;—not round in the convex part, but in the first part of the bending a little broad, and in the outward sharp-pointed end lancet-shaped; in both edges fully rounded and blunt. It was introduced into the anterior chamber, so as that the edges of the point were directed upwards, and those of the handle downwards: the point itself, however, which was turned neither towards the iris nor the cornea, he pushed suddenly through the anterior chamber to within a line's breadth of and above the still perceptible pupillary margin, towards the angle of the eye; then, without injuring the iris, he turned the instrument so that the sharp point immediately touched its internal and upper part, and, gliding perpendicularly downwards upon the anterior surface, made a superficial incision of the length of the half of the entire breadth of the anterior chamber; in which operation so little pressure was applied, that the iris was only incised, not cut through. After this incision, the instrument was turned backwards from below upwards to the middle of the incision: the iris was pierced through with the point. The instrument was then withdrawn somewhat towards the point of entrance; through which manœuvre a tolerably long, but, on account of the lying behind cataractous lens, irregular, unequal, slit in the iris was produced. The instrument was now, with the edges of the pointed end upwards, and with those in the handle downwards, carried in a horizontal direction again along the sort of engravure in the iris, and afterwards into the incision made in its substance. It was pushed forwards and inwards, nearly half a line farther, towards the internal angle; and, without minding the iris set free, so turned, that the edge of the lens, which is directed towards the internal angle of the eye, could be seized fast with it. Now the instrument was so turned that the pointed edges were situated half inwards and upwards, those on the handle half outwards and downwards, and carried backwards towards the corneal opening, by which the whole lens was pulled, with its perpendicular axis, into the newly-formed pupil, and in such way that the internal margin of the lens was before, the external behind, and the posterior surface turned towards the inner angle of the eye, the anterior towards the external. Dr. Weller attempted, by varied movements of the instrument, to tear as much as possible the cataractous substance, without thereby forcing the lens out of the newly-formed pupil; and then drew out the needle, with the edges directed in such a way as not to lacerate the wound in the cornea.

"The new pupil was apparently large, oval-shaped, and its situation in the middle of the iris, though rather somewhat turned towards the internal angle. The cataract stretched so far into the anterior chamber as almost to touch the cornea; the bleeding of the iris was not remarkable. On account of head-aches which supervened, and also of sparks of fire seen by the patient, a proper quantity of blood was taken from the arm, and ten leeches applied, which removed those symptoms.—Morning and evening, a grain of calomel was administered, and the most severe regimen observed. In thirteen days, the absorption was remarkable; three weeks after the operation, there could only be seen a thin tissue in the pupil. In other fourteen days, even this was removed, and the pupil appeared fully black. The patient could distinguish colours only tolerably, could see the window, and perceive a man standing before her, as through a mist, because the retina had been injured by the inflammatory process which had preceded the operation."

We presume to give no opinion on this case: indeed, it is with difficulty that we are able to pursue the surgeon through the various steps of the operation; and we feel almost inclined to say, with old Gobbo, when puzzled by the direction to the Jew's house, "'Twill be a hard way to hit."

A Report has been made by Baron PERCY to the French Institute, upon a new instrument *(kystitome caché,)* for the extraction of the cataract, invented by Dr. BANCAL. This report is altogether highly in favour of the invention, or rather the alteration of the instrument as formerly employed by LAFAYE, and described in the memoirs of the Academy of Surgery. The inconveniences of Lafaye's instrument were, that it was difficult to fix it steadily in the hand, from the roundness of the handle; it operated from below upwards, which rendered it less manageable; and there were no means of graduating or proportioning the length of the blade which should issue from the sheath: besides which, it required some degree of force to produce that effect, and consequently a vacillation was produced, highly necessary to be avoided among parts of so delicate a structure. These inconveniences are got rid of in M. Bancal's instrument, of which we lately presented our readers with a description.* The commissioners say that this instrument performs its office admirably. It is held like a writing-pen, and is as easily managed. "We are persuaded, (they add,) that in all those cases where it is necessary to disengage the crystalline lens from all its attachments, to divide and to open the firm and thick capsule that confines it, no instrument can be superior to that of M. Bancal; the use of which will be established and extended by the result of some trials lately made at Paris, confirming the satisfactory account of those which took place at Bourdeaux."

Professor THAL,† of Copenhagen, has invented a *rotation saw,* but of which we shall not be able to give a very intelligible account without the assistance of an engraving. The following description will, perhaps, convey some idea of the construction and powers of this instrument:

"To make a short, deep, straight incision, with a saw, in a large flat bone, by the instruments at present in use, or with which I am acquainted, if not impossible, is at least a very difficult task. One which, in my judgment, is more suitable for this purpose, is constructed in the following manner:

"1. A piece of thick watch-spring steel is formed into a circular plate or segment of a circle; its edge is furnished with coarser or finer teeth, which resemble in figure an isosceles triangle, with the angle at the apex equal to sixty degrees.

---

* *Revue Medicale,* Fevrier.—*London Med. and Physical Journal,* May.

† *Edinburgh Med. and Surg. Journal,* January 1823.

"2. The saw is fixed at right angles in the middle point (centre) from its cutting edge, to a steel rod of indefinite length, the opposite end of which has a handle like that of the trephine.

"3. Near the saw, the steel rod passes through a metallic cylinder or box, which is fixed in a flat wooden handle, perpendicular to the steel rod.

"4. According to circumstances, there are several saw-blades, commonly circular plates or segments, the teeth of which are fewer or more numerous, larger or smaller.

"The manner in which I myself use this instrument, which may be named a rotation-saw, is as follows:—I allow the handle of the brass box to rest on the fore-finger of the left hand, and with the thumb and other fingers I hold it steady, while I fix the saw on the bone which is to be divided, and make rotation with the right,—either with the whole hand, when this is practicable, or with the thumb, fore and middle finger, where there is not room for the whole hand. Each saw, therefore, may also be screwed on, parallel or perpendicular to the direction of the rotation-handle.

"The expedition with which this instrument, after a little practice, works, cannot be accurately determined. I cut through, in the presence of the secretary of our Society and others, on a subject having middle-sized bones,

| | |
|---|---|
| A cranium $2\frac{1}{2}$ lines thick, in . . . . . . . | 15 sec. |
| The ulna transversely near the carpus, in . . | 15 sec. |
| The metacarpal bone of the middle finger, in | 18 sec. |
| The ulna longitudinally near the carpus, in . | 15 sec. |

In nearly the same time I have cut through a clavicle and a rib, by this instrument, which may be used even on a hollow flat bone."

We may observe, that the Editor of the Edinburgh Journal, who is in possession of one of these saws, says, "that, though not so dexterous in its use as Professor Thal, we have found it work well; and that, to use it with effect, the fixed handle must be kept very steady, and the rotation handle moved lightly."

When we come to enumerate the various works, both English and foreign, relating more particularly to surgery, and which have been published within the last half-year, we find little to congratulate ourselves upon. Among the most prominent of those published in our own country, is a volume upon *Diseases of the Urine and Urinary Organs*, by Mr. Howship, and of which we shall, in an early Number, attempt an analysis:—we say attempt, because the work is, from the circumstance of its doctrines being almost in every instance illustrated, and explained principally, by cases, by no means an easy task for a reviewer. A little treatise on *Operative Surgery* has also been published by Mr. Averill,* the execution of which is highly respectable: it contains a great deal which is very modern, especially some of the modes of operating adopted in France, and will most undoubtedly be a very useful work to students, for whom it is principally designed.

* We shall give a more extended account of this work in our next Number.

Of Mr. BRIGGS' translation of SCARPA on *Cancer*, we have already expressed our approbation, and given a detailed account.—This is a meagre catalogue indeed, but, with the exception of a second edition of Sir A. COOPER'S work on *Dislocations,* it comprises every thing of importance that we have met with. The foreign publications are—two treatises on *Hospital Gangrene*, one by M. RIBERI, of Turin, the other by M. OLLIVIER; a systematic work on *Acoustic Surgery*, by M. ITARD; and a treatise by M. DUCAMP, of Paris, on *Strictures and Retention of Urine.*

There is a great coincidence of opinion in the two writers upon Hospital Gangrene: they, in the main points, agree with a countryman of our own (Mr. BLACKADER), who wrote a very sensible work on this disease, and whose statements and opinions are in a great measure confirmed by the authors above mentioned. Instead of the mineral acids, MM. Ollivier and Riberi strongly advocate the use of the actual cautery.

We shall do little more than mention M. Itard's work on the Diseases of the Organ of Hearing. The principal point of interest in these volumes is the proposal to inject the tympanal cavity, and which may either be done by opening into the mastoid cells, through the membrane of the tympanum, or through the eustachian tube: the first is not considered safe; the second is, according to our author, the only method likely to succeed in cases of congenital deafness. He employs simply warm water, but the injection is repeated ten or twelve times a-day. There is at first usually a good deal of pain produced, and other unpleasant symptoms; but, when the tube begins to be freed from its obstructions, which is known by the water escaping by the throat, the patient gradually recovers his hearing. The method of injecting the eustachian tube is next described: it is evident, however, from the details that this is an operation of much nicety, and that numerous trials must be made upon the living and dead body, before it can be practised with any thing like a prospect of success. Upon the whole, we hope that we do not pass too severe a judgment upon M. Itard's work, by saying that it does not materially advance our knowledge of the subject of which it treats.

M. Ducamp's treatise on Strictures, &c. we consider as a much more valuable book in France than it is likely to prove on our side of the Channel; since, with the exception of his particular method of applying caustic to the urethra, and his subsequent employment of dilators, there is not much but what is to be met with in the writings and the practice of the best English surgeons. In saying this, we hope we are rather adding to than detracting from the merit of M. Ducamp,* whose work evinces great research, a thorough acquaintance with this branch of English surgery, and is calculated to do much good, in his own country especially. On the subject of dilatable stricture, indeed, English surgeons are ever still apt to be misled. The disease is one of the commonest affections of the male urethra: it is the foundation, if neglected,

---

* We were sorry to notice the death of this promising young man in a late Number of a foreign Journal.

of permanent stricture; and the symptoms are often so equivocal, and so easily mistaken for slight attacks of gonorrhœa,—particularly as a little purulent discharge, coming on after connexion, is one of the most frequent symptoms,—that it too commonly happens to be overlooked, and the discharge is stopped by cubebs, copaiba, or some astringent injection; either of which remedies will, most commonly, produce a calm or a cessation of the discharge. Hence so much contest as to the diverse treatment of gonorrhœa; hence so much contradictory evidence as to the value of one medicine above another, in the treatment of that disease. The real source of the evil, meanwhile, will be found in the urethra: it is cured only by the use of metallic bougies; and under their use the discharge ceases, the unpleasant frequency of making water disappears, and the whole man, before languid and debilitated, appears to revive.

We had hoped to have had an opportunity of saying something relative to *diseases of the spine;* but, as Mr. SHAW's work has not yet made its appearance, we shall postpone our remarks until our next Retrospect, when we shall make some observations upon that subject generally, including Dr. HARRISON's cases and plan of cure.

We are in possession of some additional testimonies in favour of *iodine* as a remedy in *bronchocele,* and which we intend to lay before our readers in the ensuing Number of this Journal; though we fear that, with the exception of that complaint, its internal exhibition has not been attended with the advantages anticipated from its employment in other forms of glandular disease.

### MIDWIFERY.

Little of novelty has occurred in this department; the only publications worthy of notice relative to this branch of our profession being those of Drs. CAMPBELL and MACKINTOSH on *puerperal fever,* to which we have already referred, and in which the antiphlogistic treatment of the disease is very strongly urged.

Various detached cases, remarkable rather for their singularity than their practical importance, have been given to the public: one of these consists in an account of the rupture of the uterus and rectum, followed by parturition through the anus, which is described in the London Medical Repository for March.

The only other case worthy of notice occurs in the same Journal for June. A lady, thirty-eight years of age, and of full habit, had been affected for five years with symptoms referrible to the uterus, and which had excited apprehensions of cancerous disease of that organ. She had been many years without children, but became pregnant in consequence of a second marriage. Mr. PAINTER, by whom the case is related, was called to see her in November, in consequence of pain in the loins, extending round the abdomen; head-ache, restlessness, and irritability of the stomach. She had a troublesome cough, which gave her great pain, with a sensation "as if something would force itself away, and tear her inside to pieces." There was some mucous discharge from the vagina, which was so thickened and altered as to render an examination extremely difficult. This, however, was at length accomplished, and a tumor, rather larger than a goose's egg, was discovered behind the os uteri, " high up, and rather to the left side." What follows we give in the words of the author.

" By the latter end of February, she had so far recovered as to be able to visit her friends, and on one occasion ventured to ride, in a stage-coach, the distance of ten or fifteen miles, without material inconvenience, till a morning or two after her return, when she was found by her servant in a swoon, lying on the floor: she soon recovered, and therefore did not take particular notice of the occurrence. On the 10th of March she dined with some friends in the city, three miles from her residence, came home early in the evening, and took a light supper. The morning following, I was called in haste to see her: she was on the sofa in a fainting-fit, from which she was with difficulty restored; at every attempt to raise her, she swooned afresh. As soon as she was sufficiently recovered, she was put to bed, when she complained of violent throbbing in the pelvis, extending to every part of the abdomen, and accompanied with great thirst, sickness, fainting, a violent bearing-down pain, and a constant inclination to make water. I examined, and found the tumor occupying a much larger portion of the pelvis than formerly; but, as no fluctuation was perceptible, I could not compare it to any thing else but a collection of hardened fæces in the rectum: an examination per anum conveyed the same feeling. I ordered an emollient enema to be injected, prescribed opiates to allay the violence of her pain, and bled her, the pulse having become strong and frequent. The os uteri was not much altered since my last examination, yet she considered herself in the eighth month of pregnancy, and, fully conceiving herself in labour, desired me not to be from home: the pains were so acute, that she declared she could not survive greater agony, and that it was like tearing her to pieces to move even her limbs. The night became one of extreme anxiety and restlessness; thirst, sickness, and fainting continuing, which no remedies would relieve. I visited her early in the morning, (being the 12th,) and dispatched a messenger for Dr. Gooch. In the interim I prescribed a saline draught, and was again sent for in great haste about half-past twelve o'clock. She had been out of bed to the night-chair, where she fainted. I assisted her friends in placing her on the bed, and sent for my neighbour and friend Mr. Scott, surgeon, of Romney-street, who came directly, but not in time to see her before she expired, which took place a few minutes after I came to her assistance.

"This unforeseen event induced me, in concert with Mr. S., to propose to her friends the chance of saving the life of the infant by the Cæsarean section, which proposition Dr. Gooch, who had by this time arrived, joined us in recommending, and which being acceded to, I immediately performed.

" *Dissection.*—Having divided the abdominal parietes from the umbilicus to the pubes, a large quantity of fluid and coagulated blood escaped, to the amount of several quarts, and exposed to view a large substance, very much resembling, in size and appearance, the head of a fœtus, protruding from the external surface of the uterus; but which, on closer inspection, I found to be the larger one of two semi-cartilaginous substances, growing by small necks from near the right horn of this viscus. After removing the blood, which was diffused in every direction among the bowels, the uterus appeared perfectly entire, but irregularly and enormously enlarged. Turning a little to one side, I found the fœtus, which was lying loose among the intestines, and immediately directed my finger along the umbilical cord to the placenta, which was firmly attached within a membranous sac on the left and posterior side of the womb. I removed the fœtus, which was a female about the fifth month: it appeared to have been dead some days. I afterwards removed the uterus and parts connected with it, together with the fœtus: the whole weighed, in their exsanguineous state, upwards of six pounds. The parts concerned in this extraordinary case are preserved, and in my possession.

"At my leisure, I separated the placenta, which adhered firmly to the substance of the ovarium, and to a great part of the internal surface of the membrane which enveloped the fœtus. I afterwards made a section of the uterus, commencing an inch from the os tincæ, and carrying it through the anterior part of the fundus. This viscus was enlarged and diseased in a remarkable degree: its parietes were very greatly thickened, and in a very irregular manner, owing to the development of tumors in its texture, which appeared to originate in an infiltration of lymph, which had become organised, and presented a dense and semi-cartilaginous appearance: this had taken place chiefly towards its external surface, giving the uterus an irregular form. Two of these tumors protruded from this viscus, as was just noticed, and were attached to it by narrow pedicles. The internal surface of the uterus was regular, and presented an appearance resembling the decidua: the uterine cavity was not much enlarged. On inspecting narrowly the internal surface of the womb, in order to find the canal running from the cornua into the fallopian tubes, this passage could not be detected; and on making the examination from the fimbriated extremities, after having detached some of these fimbriæ from the adhesions they had formed, the fallopian tubes were ascertained to be quite impervious throughout. The right ovarium was entire. The fœtus was formed in the left ovarium: the duplicature of the peritoneum enclosing this organ constituted the more external covering of the fœtus; its internal one was its own proper membranes. The placenta was intimately connected with the structure of the ovarium, and appeared to be attached to it without any intervening texture. The placenta was consequently supplied from the vessels of the substance of the ovarium: no other vessels than those belonging to this viscus could be observed to be externally connected with the sac enclosing the fœtus, and those were greatly enlarged. The rupture of the enveloping membranes, which occasioned the fatal hemorrhage, had taken place on their superior and anterior sides, not far from the womb, and had torn a portion of the placenta."

This case very strikingly resembles that described by Dr. GRANVILLE in the Philosophical Transactions for 1820.

The *Secale cornutum*, or ergot of rye, which was deemed so deleterious by the French, in 1774, as to be prescribed by a legislative act, has of late attracted the notice of American physicians, having been recommended, so early as 1807, by Dr. STEARNS, of New York, as possessing certain specific powers over the uterus, " more certain than tartrite of antimony upon the stomach, or jalap upon the intestines." According to Dr. Stearns, the ergot is indicated, and may be advantageously given, under the following circumstances:

" 1. When, in lingering labours, the child has descended into the pelvis, the parts dilated and relaxed, the pains having ceased, or being too ineffectual to advance the labour, there is danger to be apprehended from delay, by exhaustion of strength and vital energy, from hemorrhage or other alarming symptoms.

" 2. When the pains are transferred from the uterus to other parts of the body, or to the whole muscular system, producing general puerperal convulsions.

" 3. When, in the early stages of pregnancy, abortion becomes inevitable, accompanied with profuse hemorrhage and feeble uterine contractions.

" 4. When the placenta is retained from a deficiency of contraction.

" 5. In patients liable to hemorrhage immediately after delivery. In such cases the ergot may be given as a preventive, a few minutes before the termination of the labour.

" 6. When hemorrhage or lochial discharges are too profuse immediately after delivery, and the uterus continues dilated and relaxed without any ability to contract."

On the other hand, we are informed that—

" 1. It should never be administered when nature is competent to a safe delivery.

" 2. It should never be administered until the regular pains have ceased, or are ineffectual, and there is danger to be apprehended from delay.

" 3. It should never be administered until the rigidity of the os tincæ has subsided, and a perfect relaxation induced.

" 4. It should never be administered in the incipient stages of labour, nor until the os tincæ is dilated to the size of a dollar.

" 5. It should never be administered in any case of preternatural presentation that will require the fœtus to be turned.

" 6. It should never be administered, during the continuance of one labour, in larger quantities than thirty grains by decoction in half a pint of water."

The ergot, when judiciously administered, is said to possess the property of exciting the uterus to contraction; and an instance is cited by the author above mentioned, in which an accoucheur, being anxious to set out upon a journey, gave the ergot to a lady whom he was engaged to attend, before any symptoms of labour had made their appearance. In an hour after, labour had begun, and proceeded through its regular stages to a safe termination. This proceeding, although it tends to illustrate the effect of the medicine, is justly condemned by Dr. Stearns as altogether unwarrantable. Under the precautions which we have quoted, the efficacy of the ergot is said to be very striking; being followed, in from five to twenty minutes after its exhibition, by a bearing-down effort, which gradually increases, and goes on without any intermission till the delivery be completed. It is this uninterrupted action of the uterus which renders the remedy so improper when the presentation is unfavourable, as any attempt to turn the child must, of necessity, prove abortive, and even dangerous.

With respect to the dose, we are informed that a table-spoonful of the decoction, (gr. xxx. to ℔ss. of water,) given every ten minutes, generally answers better than a larger dose, as it does not affect the stomach with nausea or vomiting; an operation which sometimes interrupts its action on the uterus. It is asserted that the ergot does not produce abortion, although it has frequently been given for this purpose.*

### CHEMISTRY, PHARMACY, AND BOTANY.

This ESSAY has already encroached so much on the Journal, that our remarks on these branches of science must be very laconic. The most important discovery in chemistry, is the *condensation of the gases*, by Mr. FARADAY, of which we have already given an account.† Several improvements in the preparation of various medicines have likewise been detailed,‡ chiefly taken from the French.—In botany, nothing has occurred worthy of particular remark.

* *American Medical Recorder*, No. xx.

† Number for May.

‡ See our INTELLIGENCE in various preceding Numbers.

§ Number for June.

